# Supplementary material for: New Dammarane-Type Triterpenoid Saponins from Panax notoginseng Leaves and Their Nitric Oxide Inhibitory Activities
Source: Molecules. 2019 Dec 29;25(1):139. doi: 10.3390/molecules25010139 (PMC6982892; doi:10.3390/molecules25010139)
Supplement: Supplementary file 1 [file molecules-25-00139-s001.pdf]

Supporting Information

New Dammarane-Type Triterpenoid Saponins from *Panax notoginseng* Leaves and Their Nitric Oxide Inhibitory Activities

Fan Sun [1,†], Jingya Ruan [1,†], Wei Zhao [1], Ying Zhang [2], Guilin Xiang [3], Jiejing Yan [2],Mimi Hao [2], Lijie Wu [1], Yi Zhang [1,2,*] and Tao Wang [1,2,*]

[1] Tianjin Key Laboratory of TCM Chemistry and Analysis, Tianjin University of Traditional Chinese Medicine, 10 Poyanghu Road, West Area, Tuanbo New Town, Jinghai District, Tianjin 301617, China; sf18435165322@163.com (F.S.), Ruanjy19930919@163.com (J.R.); zhaowei126123@126.com (W.Z.); wulj0816@163.com (L.W.)

[2] Institute of TCM, Tianjin University of Traditional Chinese Medicine, 10 Poyanghu Road, West Area, Tuanbo New Town, Jinghai District, Tianjin 301617, China; zyingtzy@163.com (Y.Z.); 17320072093@163.com (J.Y.); haomimi126@126.com (M.H.)

[3] WenshanMiaoxiangSanqi Limited company, South KaihuaRoad, Wenshan City 663000, Yunnan Province, China; xiangguilin@126.com

* Correspondence: zhwwxzh@tjutcm.edu.cn (Y.Z.); wangtao@tjutcm.edu.cn (T.W.); Tel./Fax: +86-22-5959-6168 (T.W.)

† These authors contributed equally to this work.

Figure S1 ^{1}H NMR (500 MHz, C_5D_5N) spectrum of **1** 4
Figure S2 ^{13}C NMR (125 MHz, C_5D_5N) spectrum of **1** 4
Figure S3 ^{1}H ^{1}H COSY (C_5D_5N) spectrum of **1** 5
Figure S4 HSQC (C_5D_5N) spectrum of **1** 6
Figure S6 HSQC-TOCSY (C_5D_5N) spectrum of **1** 6
Figure S7 ESI-Q-Orbitrap-MS spectrum of **1** 7
Figure S8 ^{1}H NMR (500 MHz, C_5D_5N) spectrum of **2** 8
Figure S9 ^{13}C NMR (125 MHz, C_5D_5N) spectrum of **2** 8
Figure S10 ^{1}H ^{1}H COSY (C_5D_5N) spectrum of **2** 9
Figure S11 HSQC (C_5D_5N) spectrum of **2** 9
Figure S12 HMBC (C_5D_5N) spectrum of **2** 10
Figure S13 HSQC-TOCSY (C_5D_5N) spectrum of **2** 10
Figure S14 ESI-Q-Orbitrap-MS spectrum of **2** 11
Figure S15 ^{1}H NMR (500 MHz, C_5D_5N) spectrum of **3** 12
Figure S16 ^{13}C NMR (125 MHz, C_5D_5N) spectrum of **3** 12
Figure S17 ^{1}H ^{1}H COSY (C_5D_5N) spectrum of **3** 13
Figure S18 HSQC (C_5D_5N) spectrum of **3** 13
Figure S19 HMBC (C_5D_5N) spectrum of **3** 14
Figure S20 HSQC-TOCSY (C_5D_5N) spectrum of **3** 14
Figure S21 ESI-Q-Orbitrap-MS spectrum of **3** 15
Figure S22 ^{1}H NMR (500 MHz, C_5D_5N) spectrum of **4** 16
Figure S23 ^{13}C NMR (125 MHz, C_5D_5N) spectrum of **4** 16
Figure S24 ^{1}H ^{1}H COSY (C_5D_5N) spectrum of **4** 17
Figure S25 HSQC (C_5D_5N) spectrum of **4** 17
Figure S26 HMBC (C_5D_5N) spectrum of **4** 18
Figure S27 NOSEY (C_5D_5N) spectrum of **4** 18
Figure S28 ESI-Q-Orbitrap-MS spectrum of **4** 19
Figure S29 ^{1}H NMR (500 MHz, C_5D_5N) spectrum of **5** 20
Figure S30 ^{13}C NMR (125 MHz, C_5D_5N) spectrum of **5** 20
Figure S31 ^{1}H ^{1}H COSY (C_5D_5N) spectrum of **5** 21
Figure S32 HSQC (C_5D_5N) spectrum of **5** 21
Figure S33 HMBC (C_5D_5N) spectrum of **5** 22
Figure S34 ESI-Q-Orbitrap-MS spectrum of **5** 22
Figure S35 ^{1}H NMR (500 MHz, C_5D_5N) spectrum of **6** 23
Figure S36 ^{13}C NMR (125 MHz, C_5D_5N) spectrum of **6** 23
Figure S37 ^{1}H ^{1}H COSY (C_5D_5N) spectrum of **6** 24
Figure S38 HSQC (C_5D_5N) spectrum of **6** 24
Figure S39 HMBC (C_5D_5N) spectrum of **6** 25
Figure S40 HSQC-TOCSY (C_5D_5N) spectrum of **6** 25
Figure S41 ESI-Q-Orbitrap-MS spectrum of **6** 26
Figure S42 ^{1}H NMR (600 MHz, C_5D_5N) spectrum of **7** 27
Figure S43 ^{13}C NMR (150 MHz, C_5D_5N) spectrum of **7** 27
Figure S44 ^{1}H ^{1}H COSY (C_5D_5N) spectrum of **7** 28

Figure S45 HSQC (C_5D_5N) spectrum of **7** 28
Figure S46 HMBC (C_5D_5N) spectrum of **7** 29
Figure S47 ESI-Q-Orbitrap-MS spectrum of **7** 29
Figure S48 ^{1}H NMR (500 MHz, C_5D_5N) spectrum of **8** 30
Figure S49 ^{13}C NMR (125 MHz, C_5D_5N) spectrum of **8** 30
Figure S50 ^{1}H ^{1}H COSY (C_5D_5N) spectrum of **8** 31
Figure S51 HSQC (C_5D_5N) spectrum of **8** 31
Figure S52 HMBC (C_5D_5N) spectrum of **8** 32
Figure S53 ESI-Q-Orbitrap-MS spectrum of **8** 32
Figure S54 ^{1}H NMR (500 MHz, C_5D_5N) spectrum of **9** 33
Figure S55 ^{13}C NMR (125 MHz, C_5D_5N) spectrum of **9** 33
Figure S56 ^{1}H ^{1}H COSY (C_5D_5N) spectrum of **9** 34
Figure S57 HSQC (C_5D_5N) spectrum of **9** 34
Figure S58 HMBC (C_5D_5N) spectrum of **9** 35
Figure S59 ESI-Q-Orbitrap-MS spectrum of **9** 35
Figure S60 ^{1}H NMR (500 MHz, C_5D_5N) spectrum of **10** 36
Figure S61 ^{13}C NMR (125 MHz, C_5D_5N) spectrum of **10** 36
Figure S62 ^{1}H ^{1}H COSY (C_5D_5N) spectrum of **10** 37
Figure S63 HSQC (C_5D_5N) spectrum of **10** 37
Figure S64 HMBC (C_5D_5N) spectrum of **10** 38
Figure S65 HSQC-TOCSY (C_5D_5N) spectrum of **10** 38
Figure S66 ESI-Q-Orbitrap-MS spectrum of **10** 39
Figure S67 ^{1}H NMR (500 MHz, C_5D_5N) spectrum of **11** 40
Figure S68 ^{13}C NMR (125 MHz, C_5D_5N) spectrum of **11** 41
Figure S69 ^{1}H ^{1}H COSY (C_5D_5N) spectrum of **11** 41
Figure S70 HSQC (C_5D_5N) spectrum of **11** 42
Figure S71 HMBC (C_5D_5N) spectrum of **11** 42
Figure S72 NOSEY (C_5D_5N) spectrum of **11** 43
Figure S73 ESI-Q-Orbitrap-MS spectrum of **11** 43
Figure S74 MTT assay of compounds **1–11** at the concentration of 50 μM........ 44

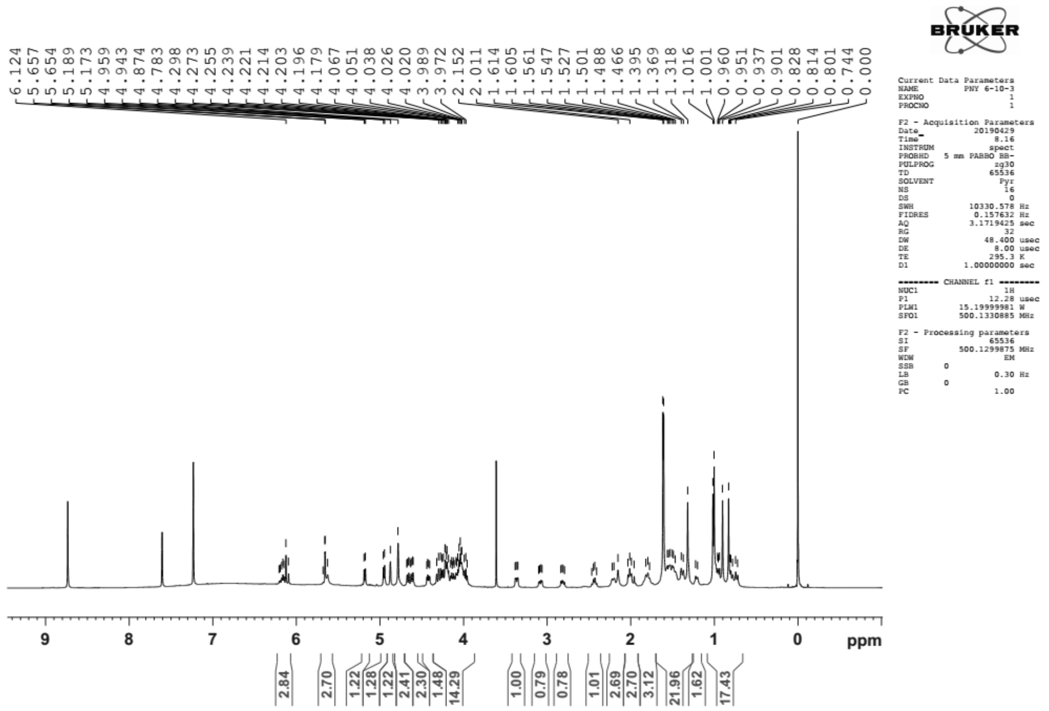

Figure S1 ^{1}H NMR (500 MHz, C_5D_5N) spectrum of **1**

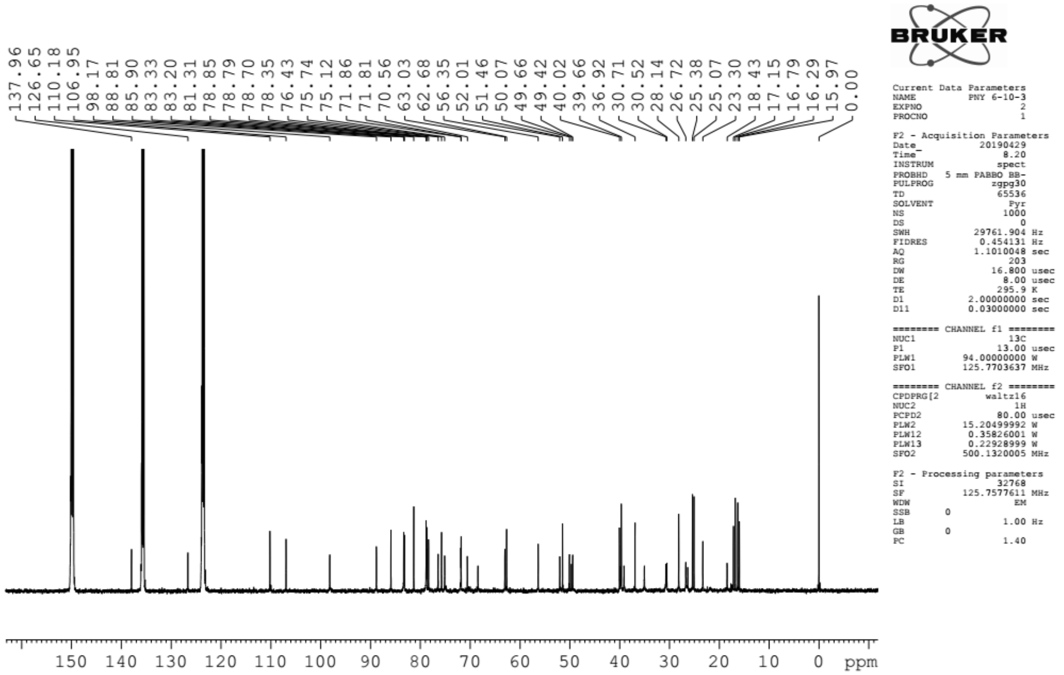

Figure S2 ^{13}C NMR (125 MHz, C_5D_5N) spectrum of **1**

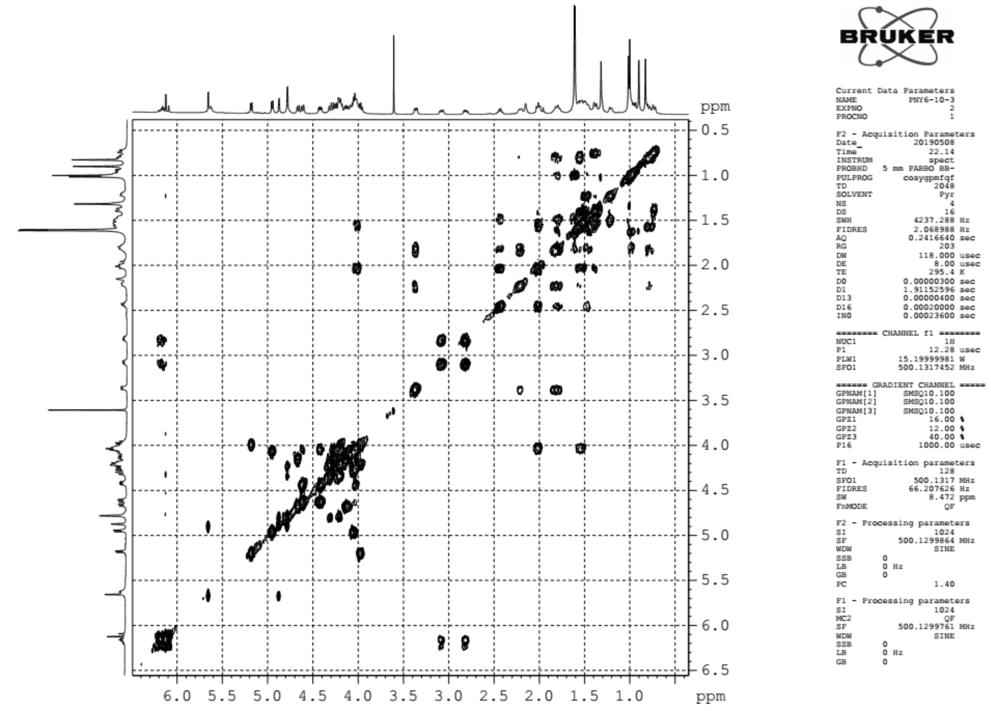

Figure S3 ^{1}H ^{1}H COSY (C_5D_5N) spectrum of **1**

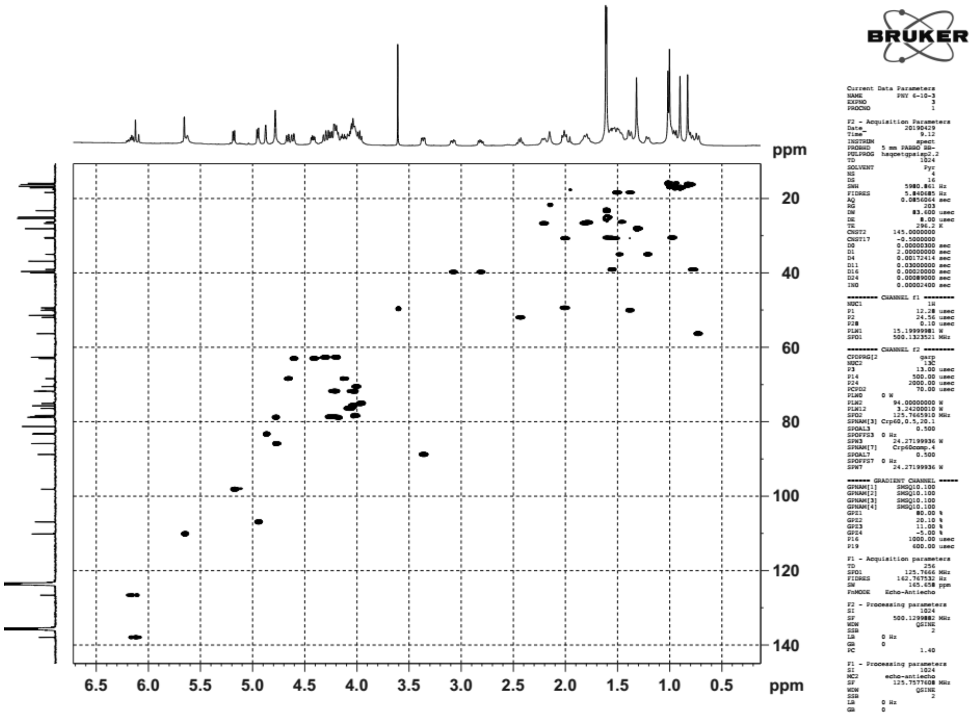

Figure S4 HSQC (C_5D_5N) spectrum of **1**

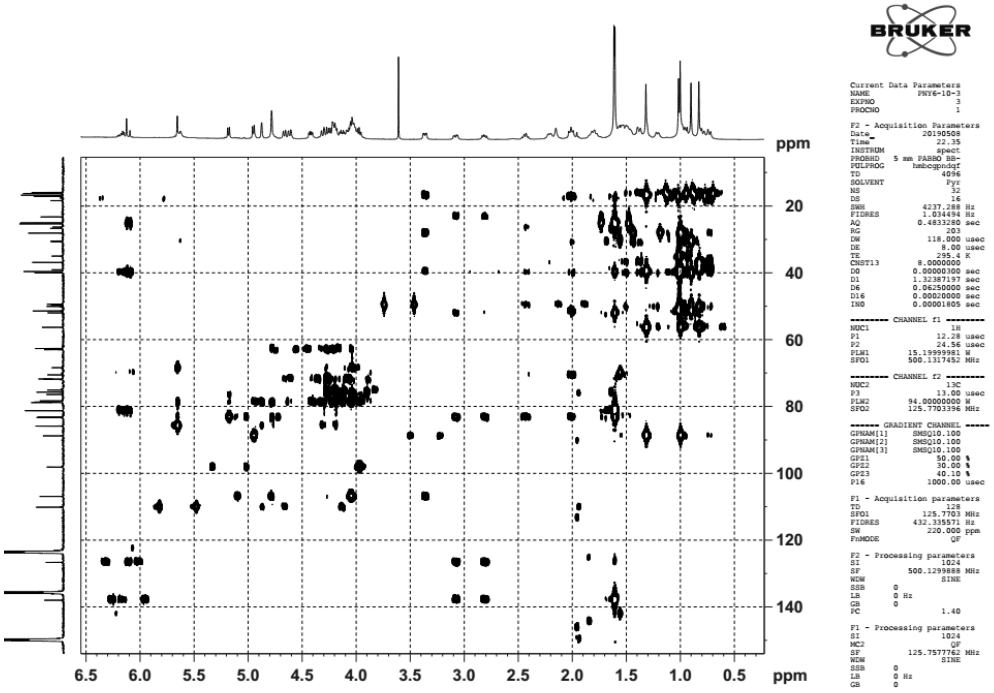

Figure S5 HMBC (C_5D_5N) spectrum of **1**

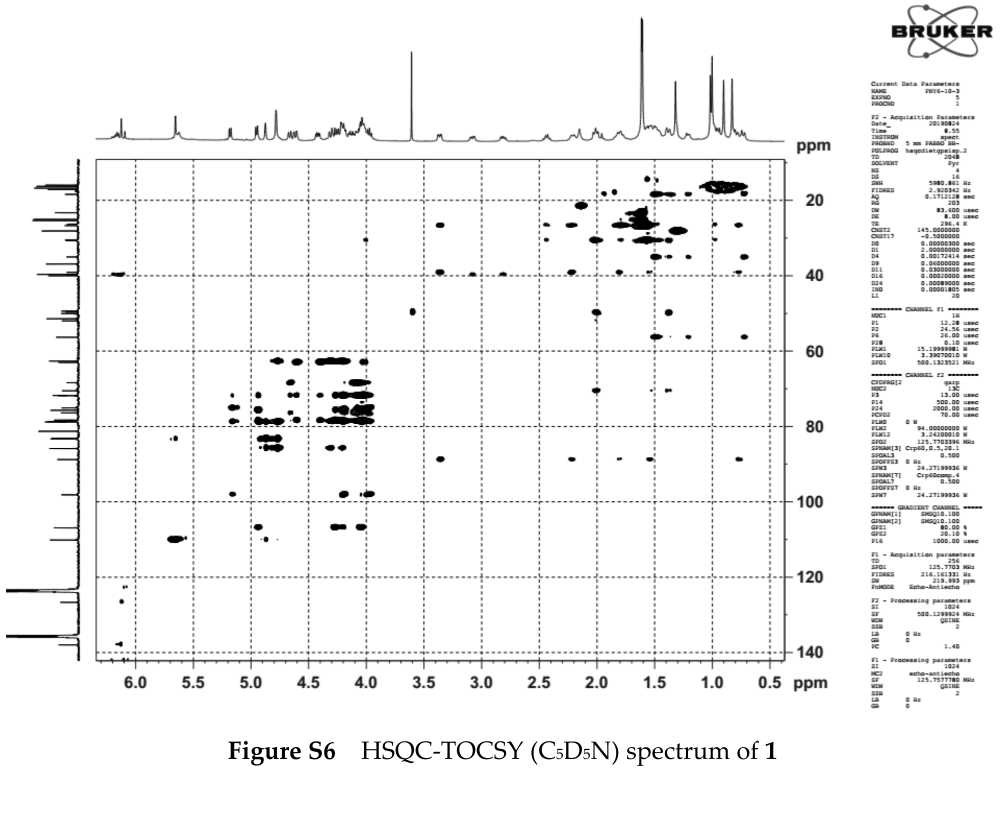

Figure S6 HSQC-TOCSY (C_5D_5N) spectrum of **1**

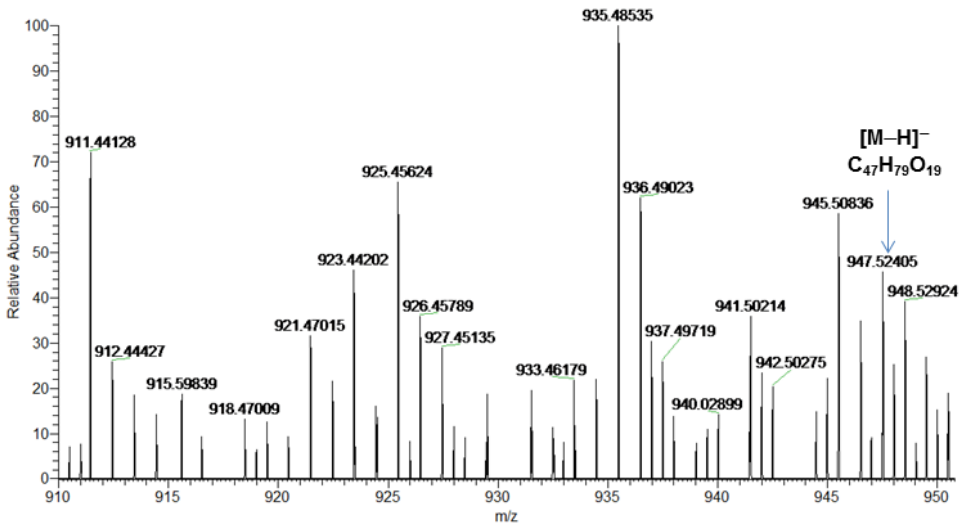

Figure S7 ESI-Q-Orbitrap-MS spectrum of **1**

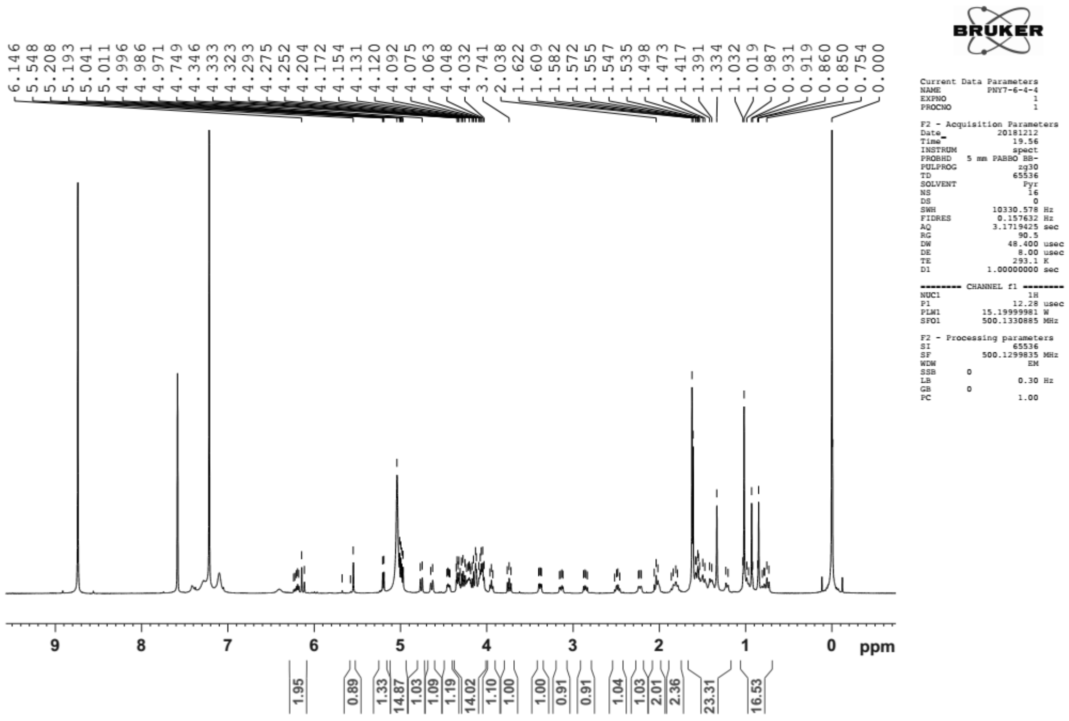

Figure S8 ^{1}H NMR (500 MHz, C_5D_5N) spectrum of **2**

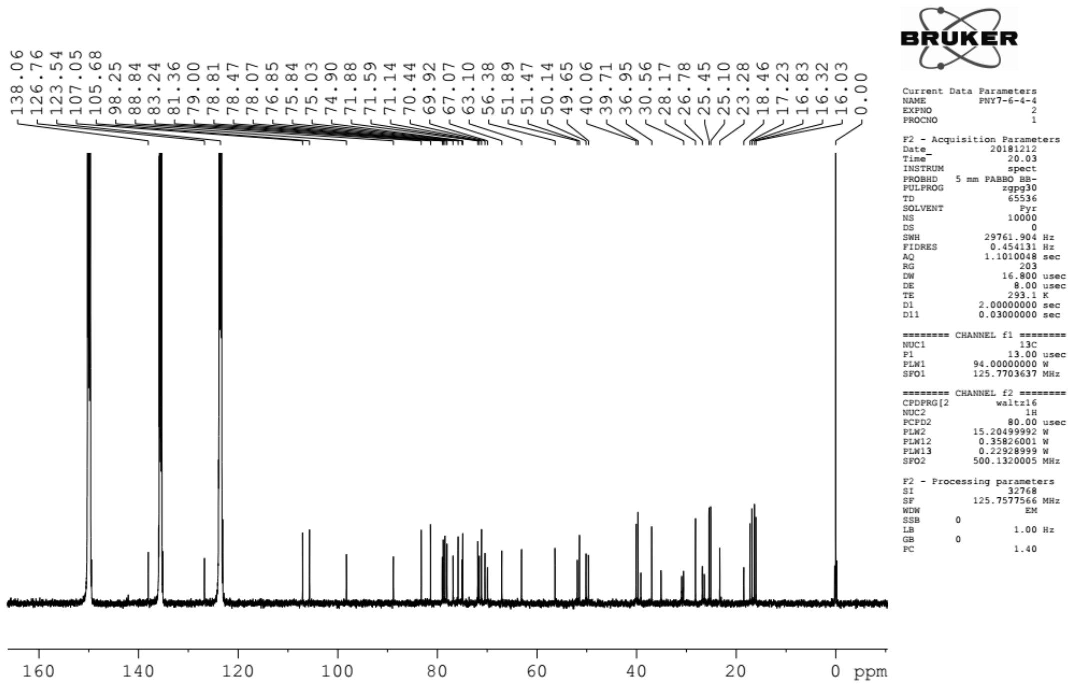

Figure S9 ^{13}C NMR (125 MHz, C_5D_5N) spectrum of **2**

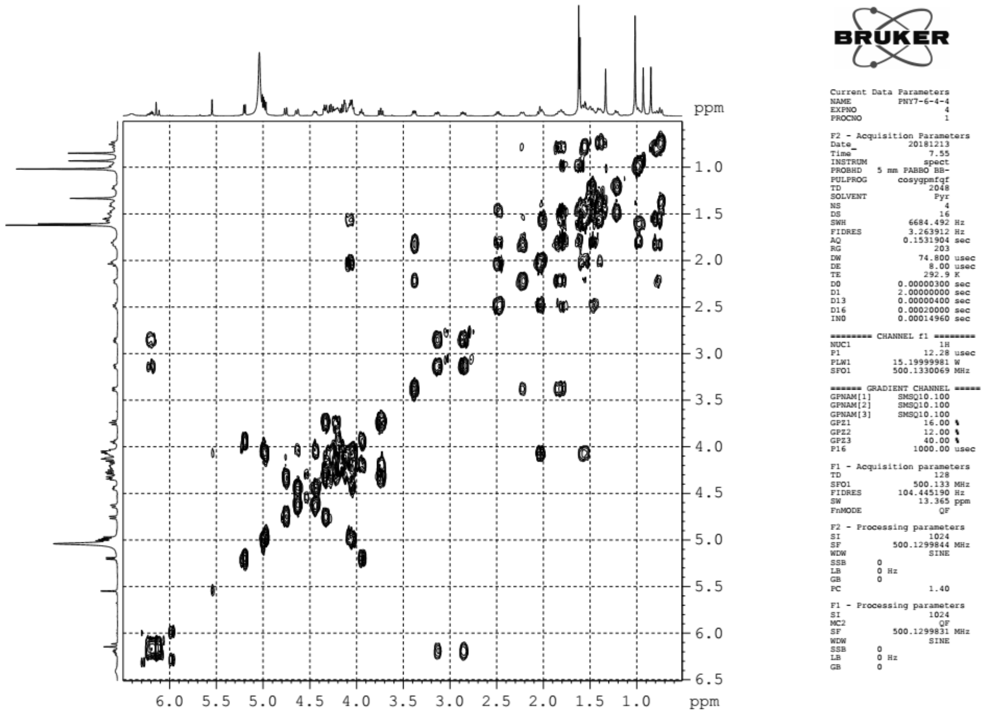

Figure S10 ^{1}H ^{1}H COSY (C_5D_5N) spectrum of **2**

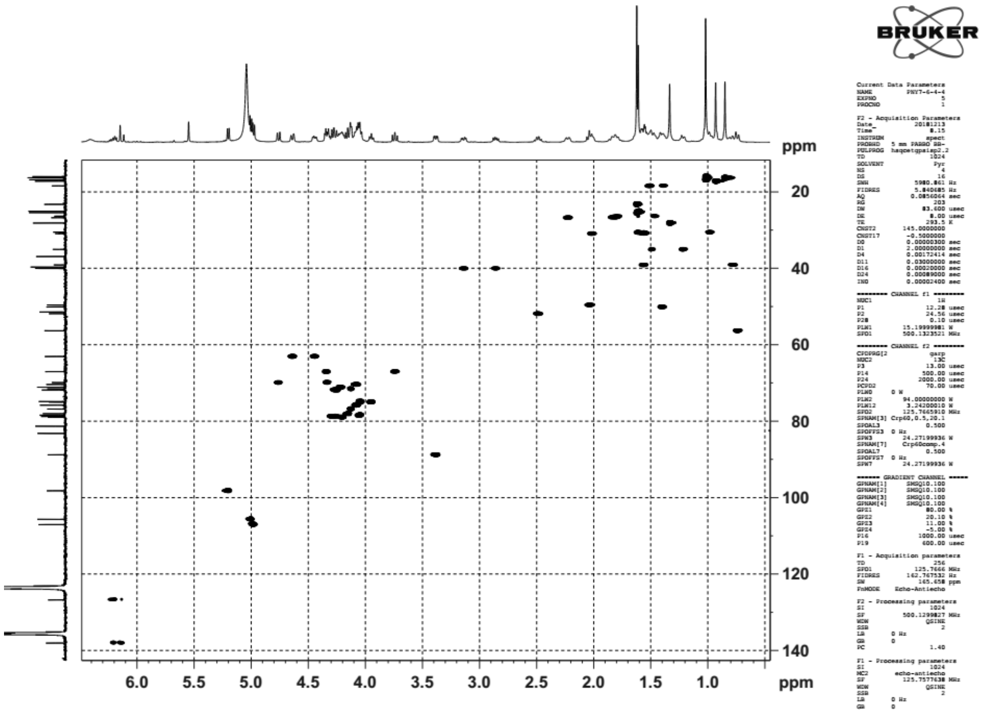

Figure S11 HSQC (C_5D_5N) spectrum of **2**

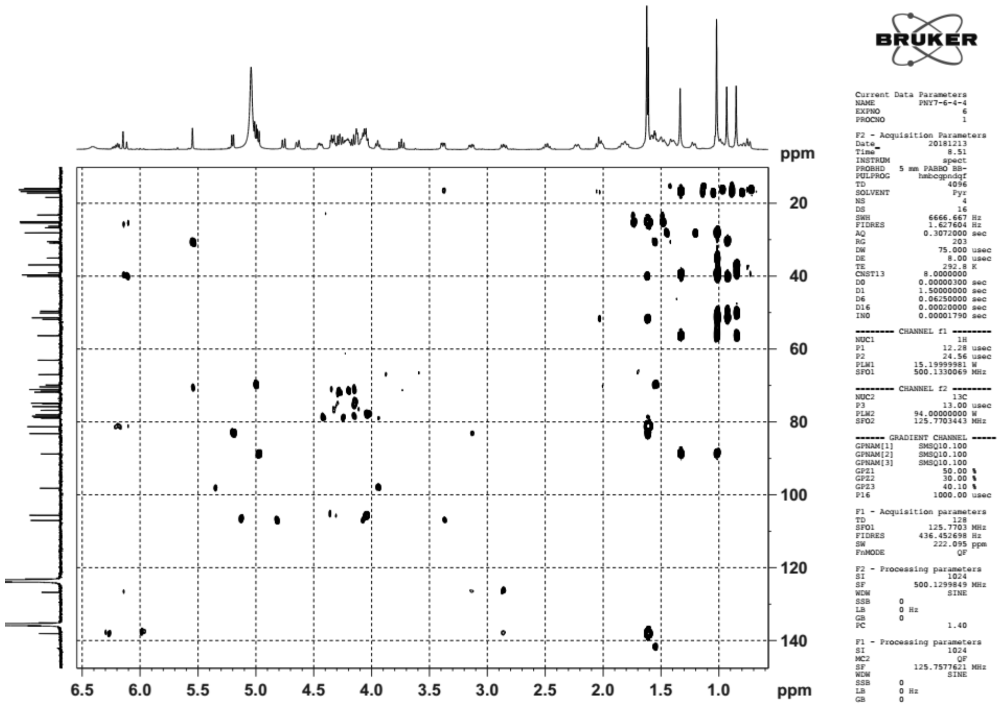

Figure S12 HMBC (C_5D_5N) spectrum of **2**

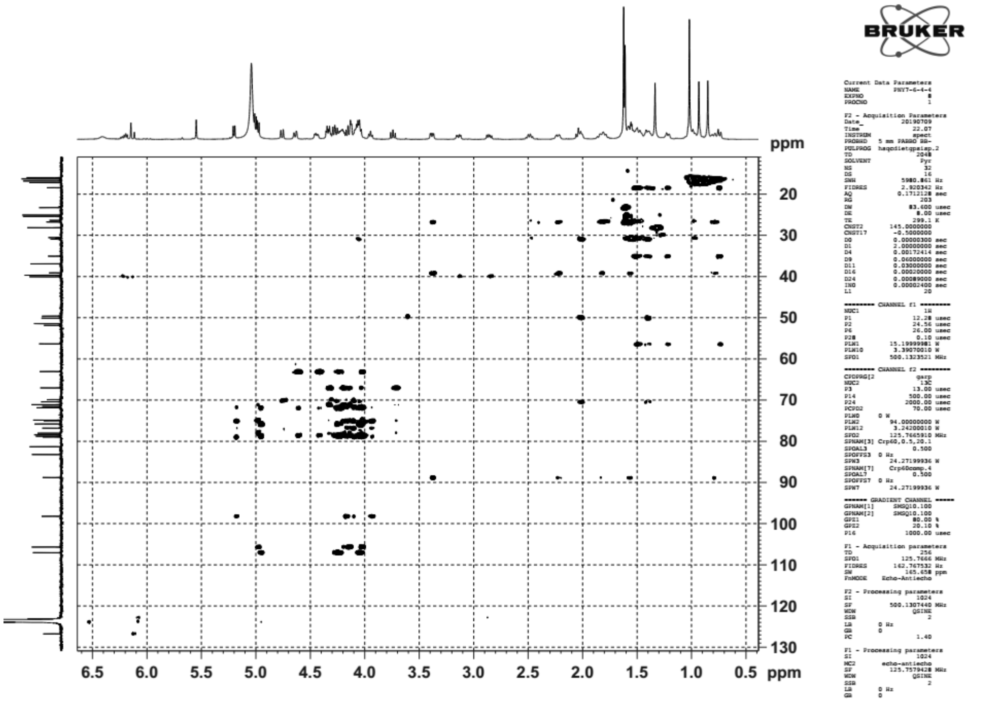

Figure S13 HSQC-TOCSY (C_5D_5N) spectrum of **2**

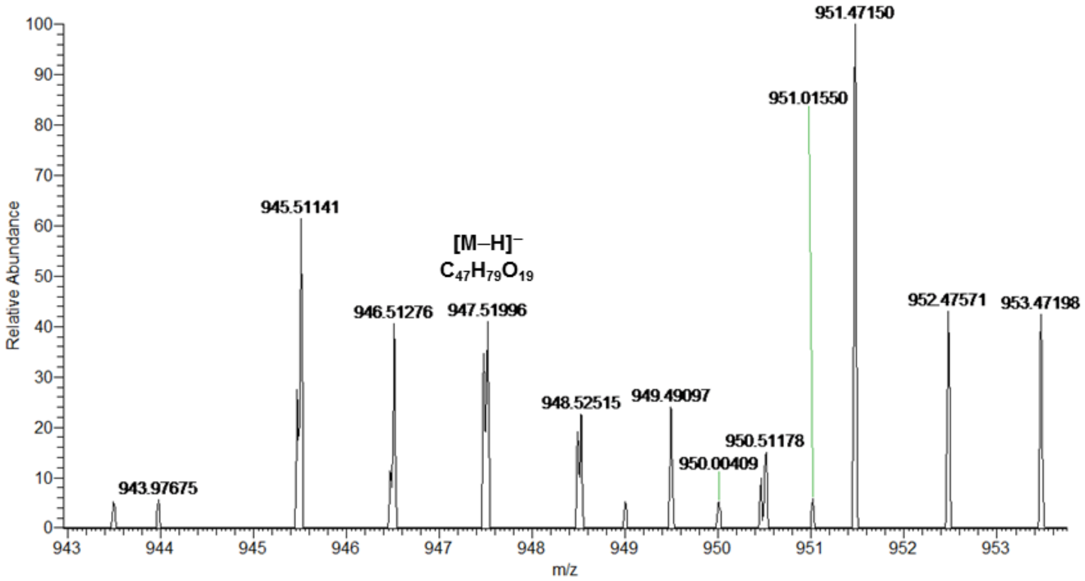

Figure S14 ESI-Q-Orbitrap-MS spectrum of **2**

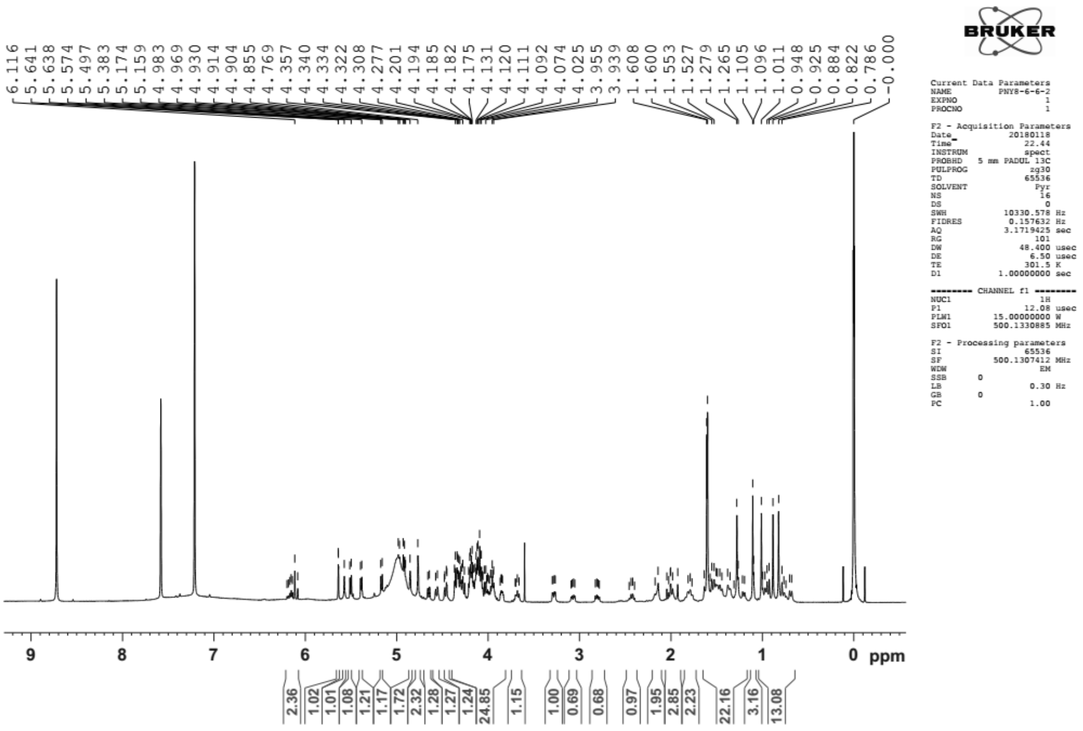

Figure S15 ^{1}H NMR (500 MHz, C_5D_5N) spectrum of **3**

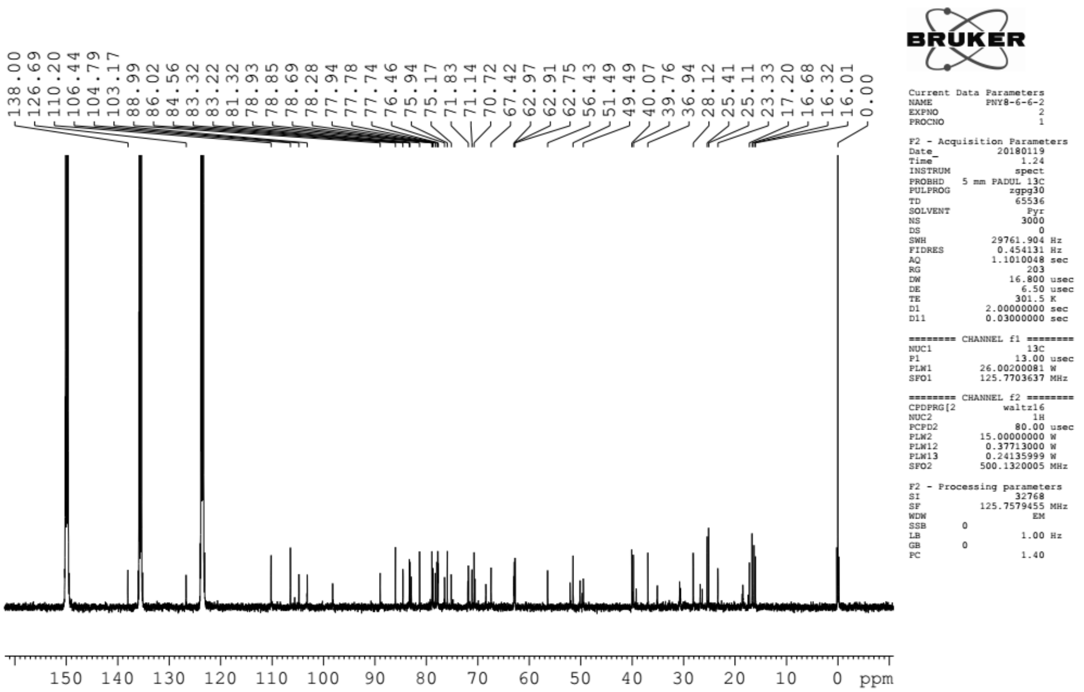

Figure S16 ^{13}C NMR (125 MHz, C_5D_5N) spectrum of **3**

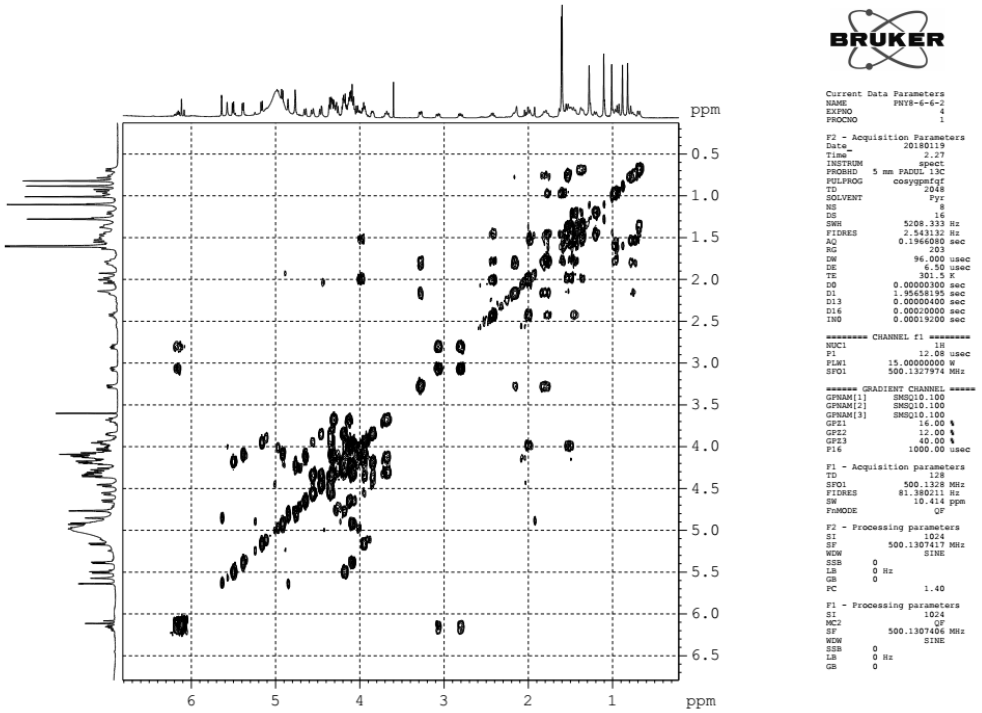

Figure S17 ^{1}H ^{1}H COSY (C_5D_5N) spectrum of **3**

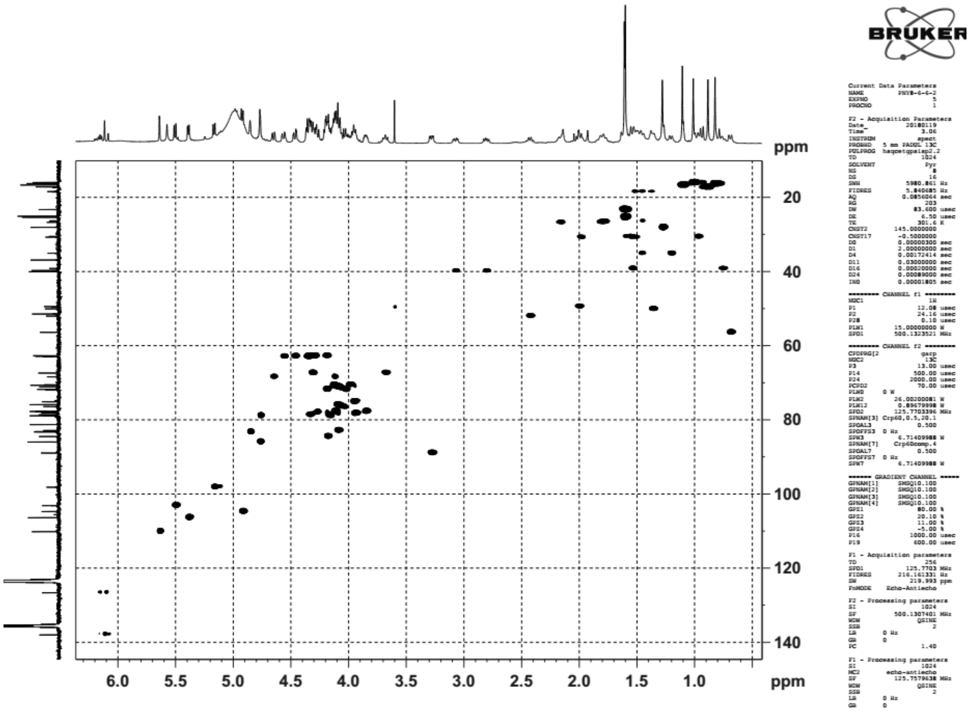

Figure S18 HSQC (C_5D_5N) spectrum of **3**

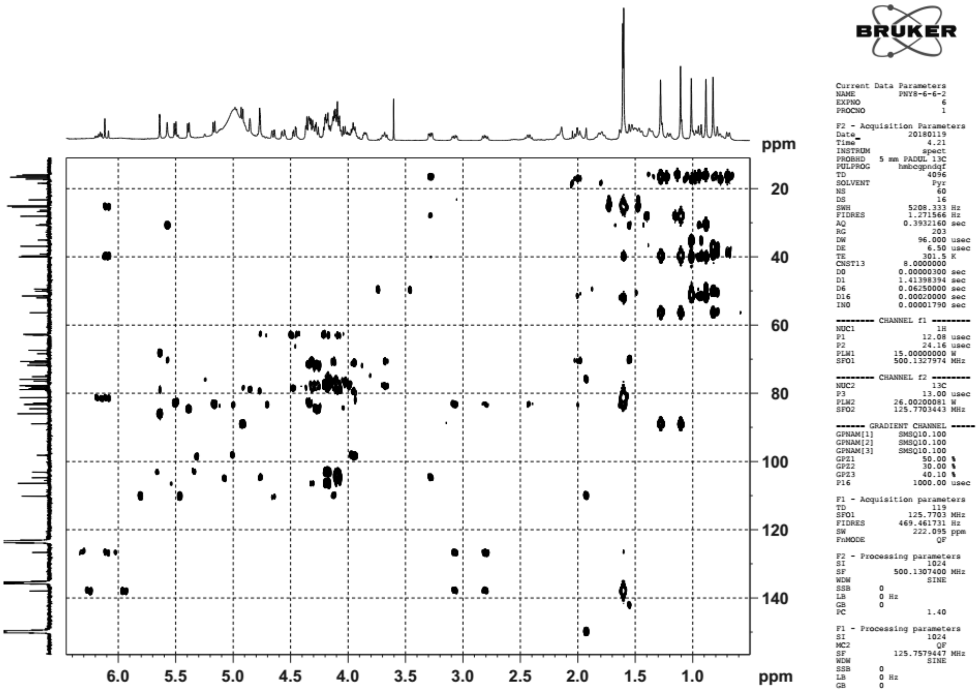

Figure S19 HMBC (C_5D_5N) spectrum of **3**

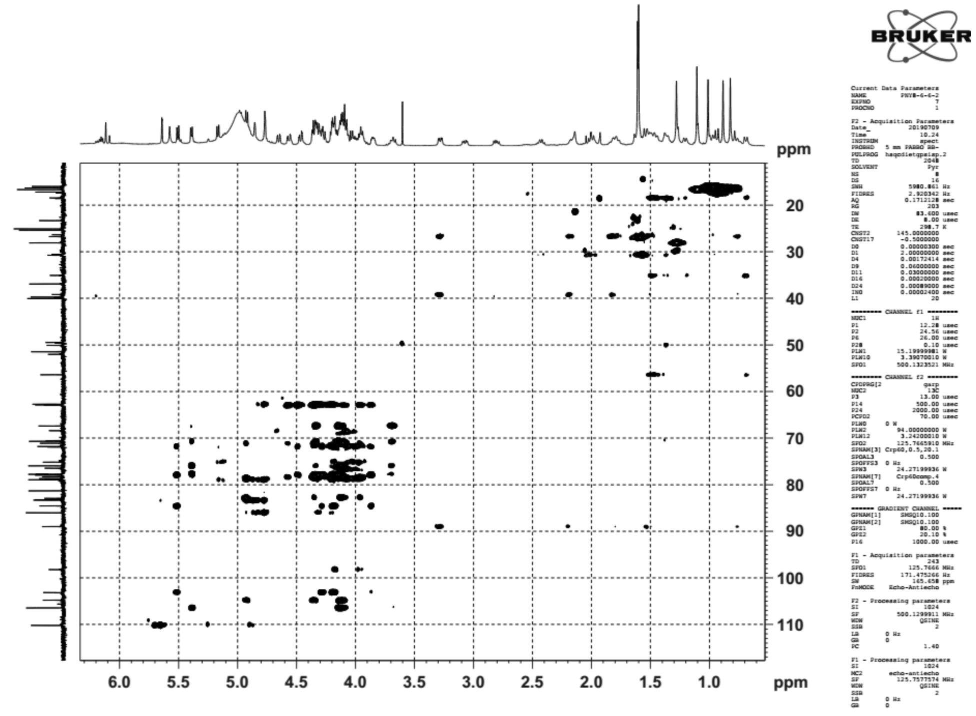

Figure S20 HSQC-TOCSY (C_5D_5N) spectrum of **3**

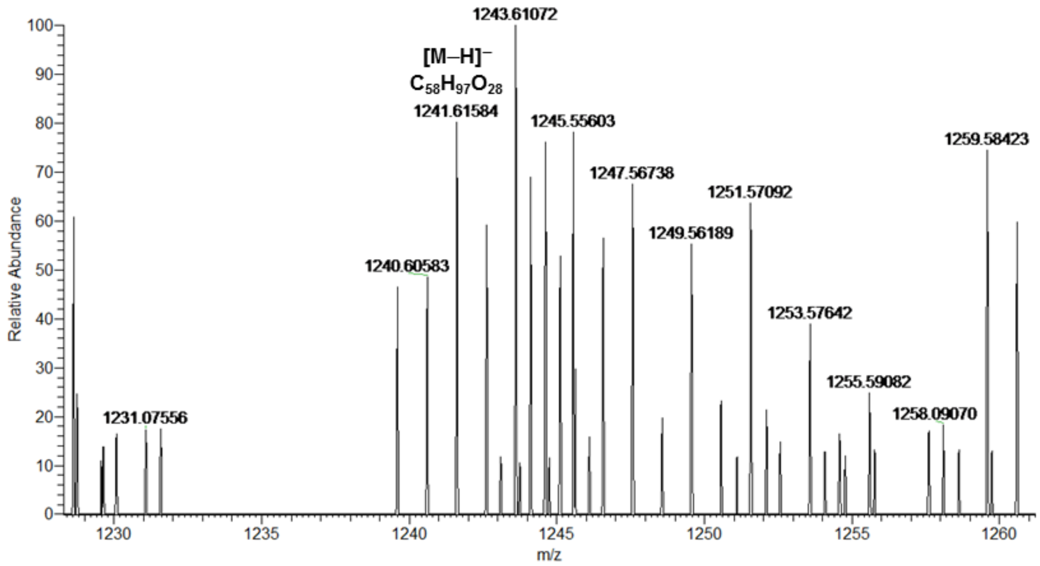

Figure S21 ESI-Q-Orbitrap-MS spectrum of **3**

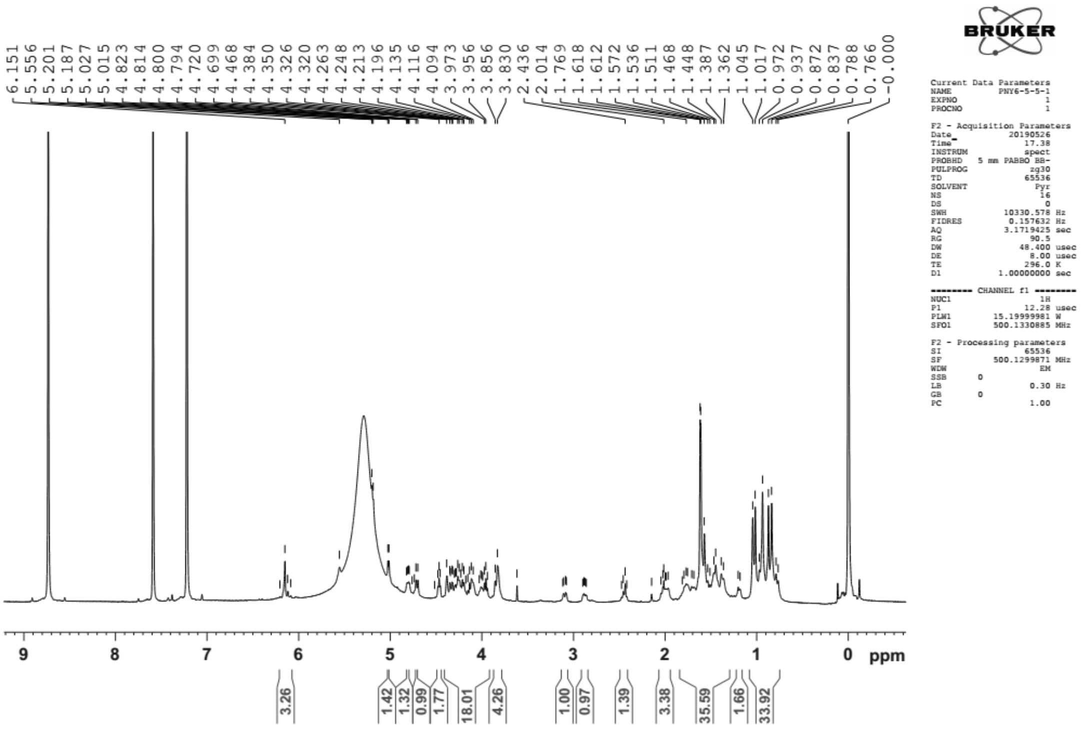

Figure S22 ^{1}H NMR (500 MHz, C_5D_5N) spectrum of **4**

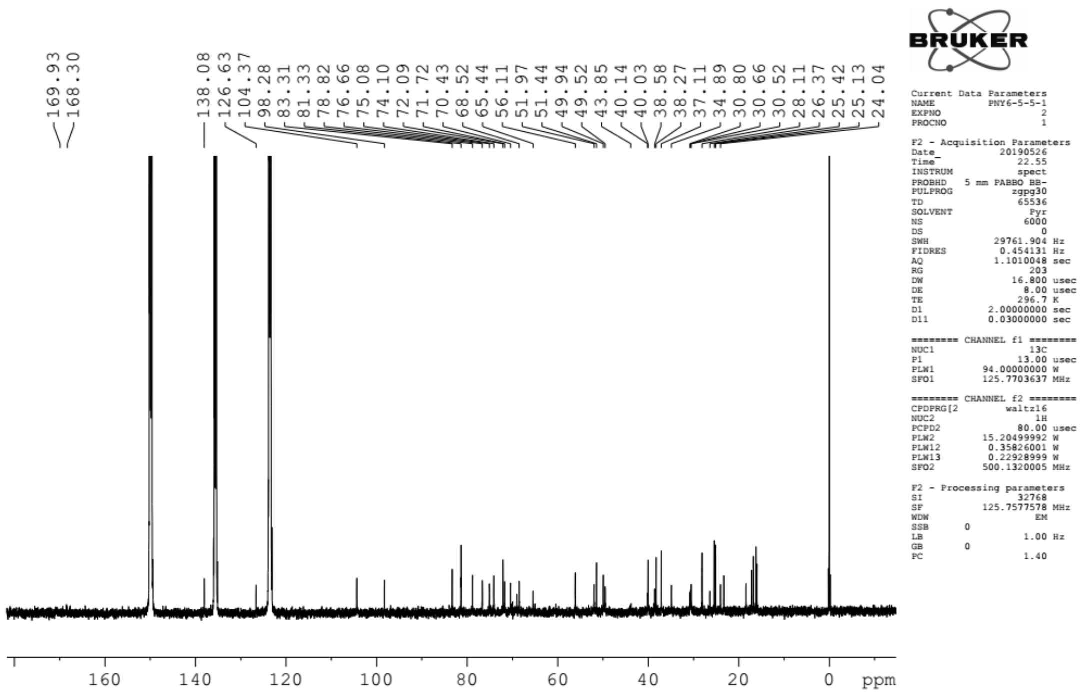

Figure S23 ^{13}C NMR (125 MHz, C_5D_5N) spectrum of **4**

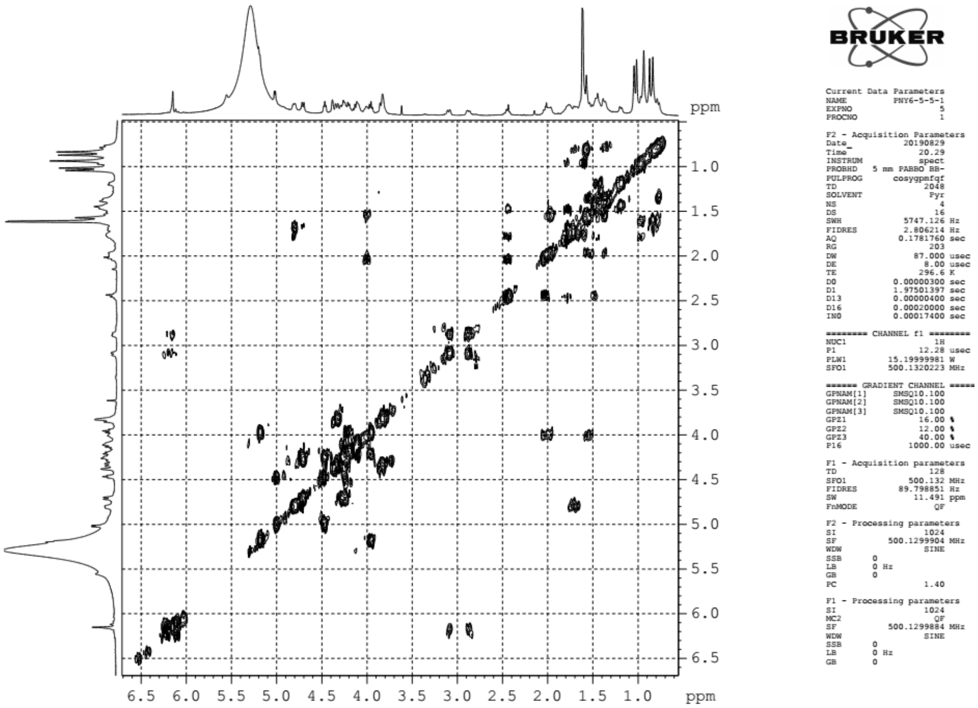

Figure S24 ^1H ^1H COSY (C_5D_5N) spectrum of **4**

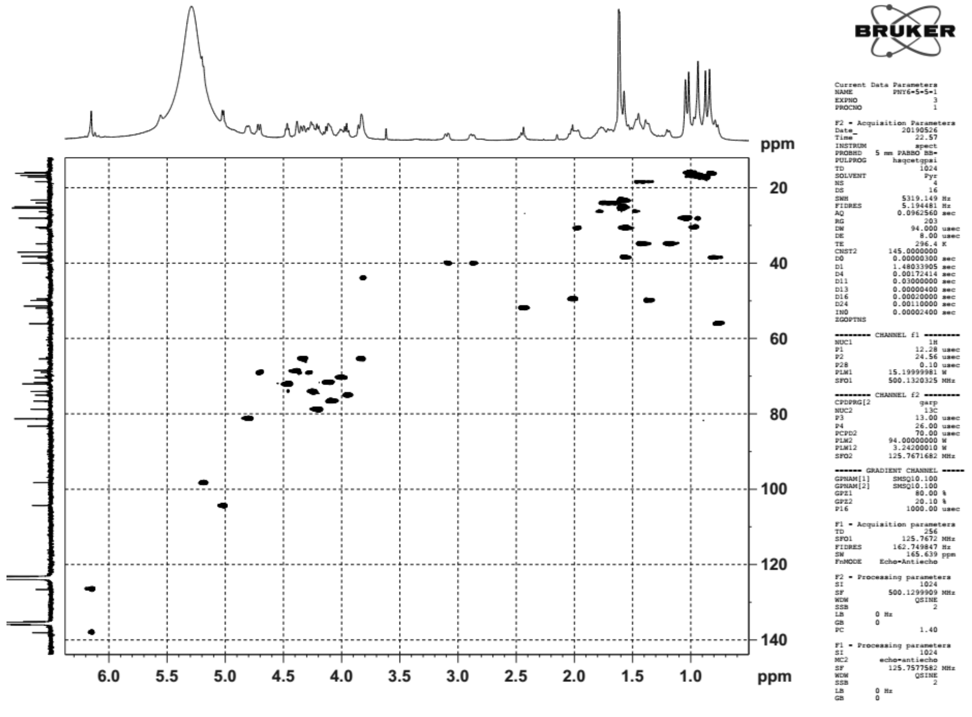

Figure S25 HSQC (C_5D_5N) spectrum of **4**

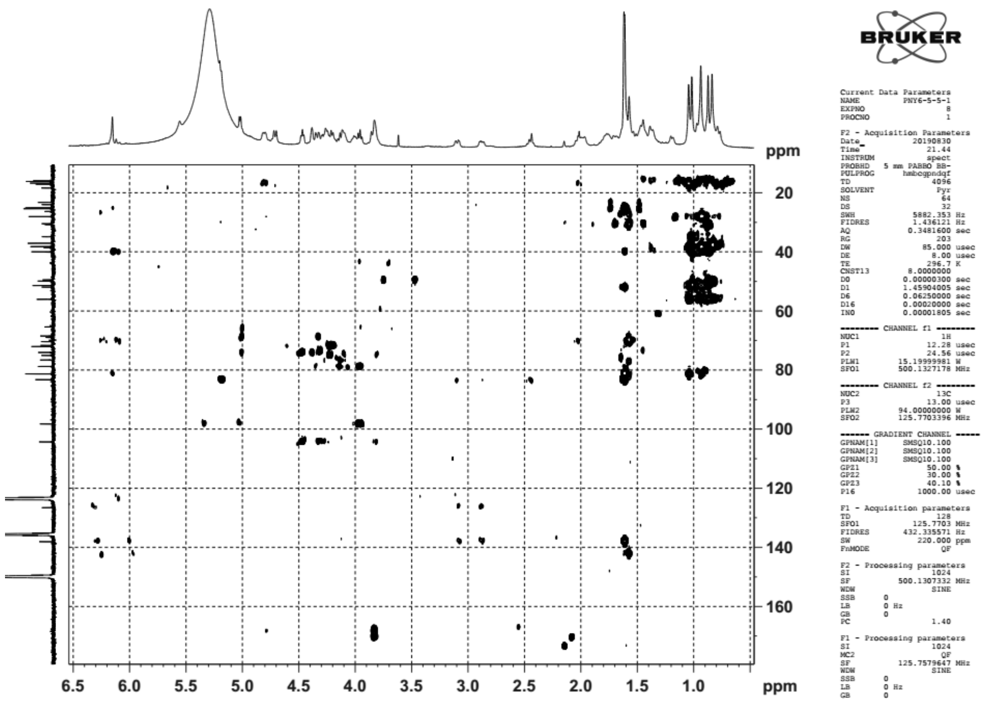

Figure S26 HMBC (C_5D_5N) spectrum of **4**

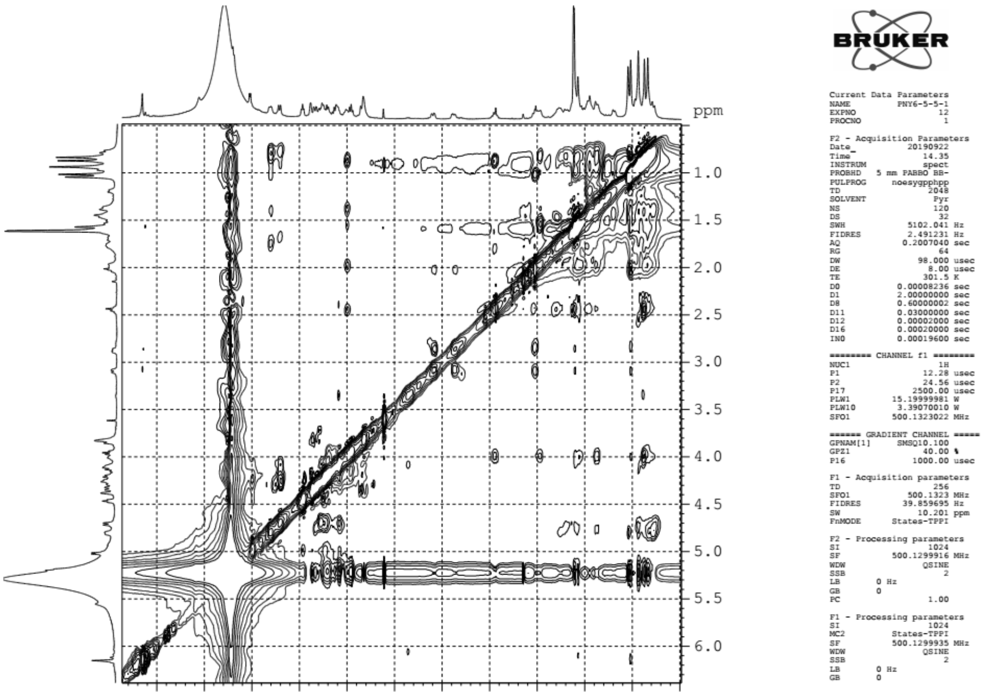

Figure S27 NOSEY (C_5D_5N) spectrum of **4**

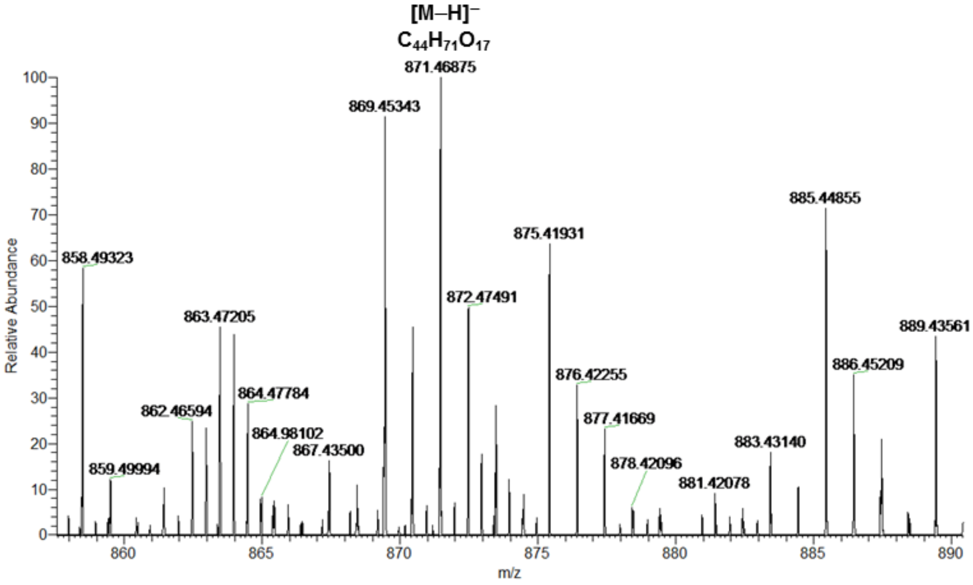

Figure S28 ESI-Q-Orbitrap-MS spectrum of **4**

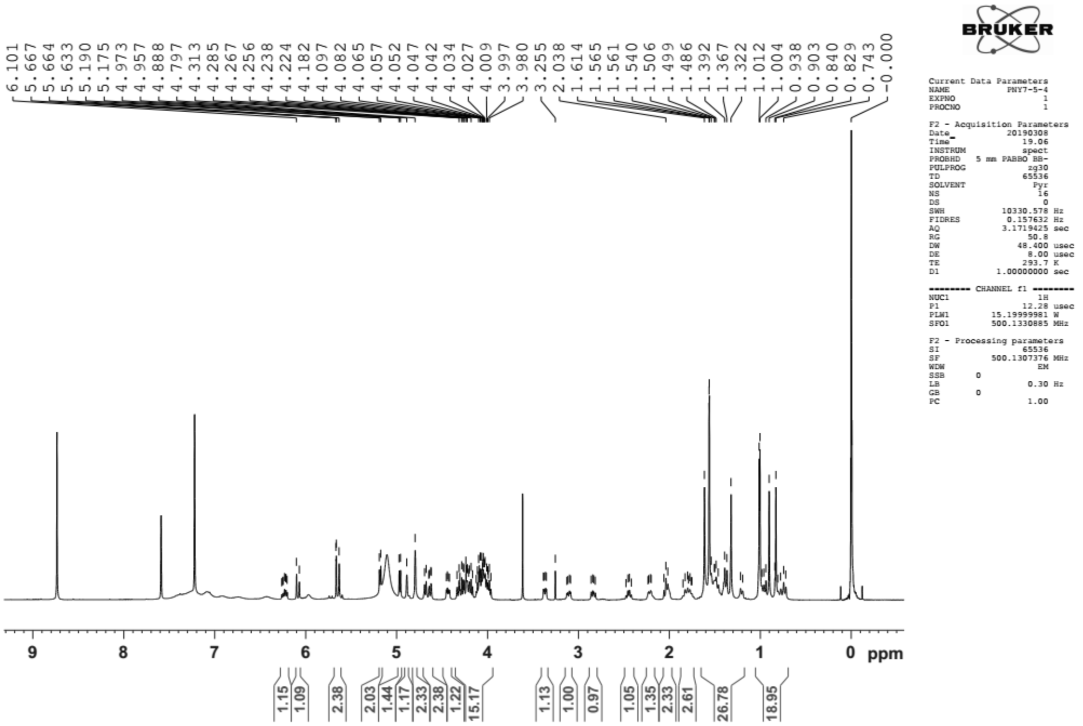

Figure S29 ^{1}H NMR (500 MHz, C_5D_5N) spectrum of **5**

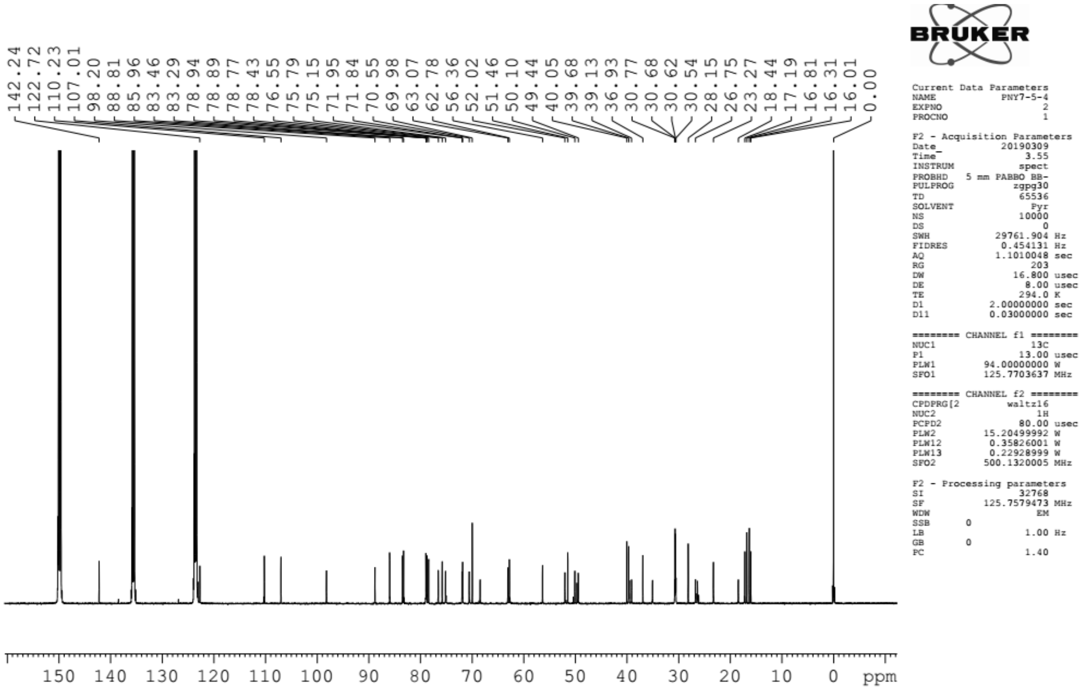

Figure S30 ^{13}C NMR (125 MHz, C_5D_5N) spectrum of **5**

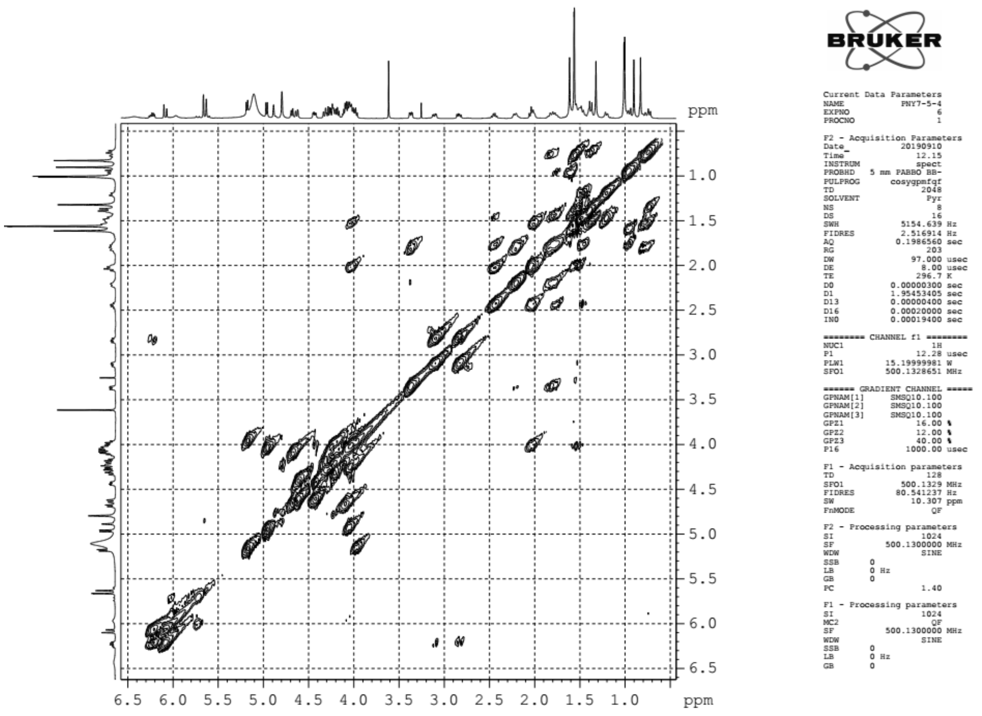

Figure S31 ^{1}H ^{1}H COSY (C_5D_5N) spectrum of **5**

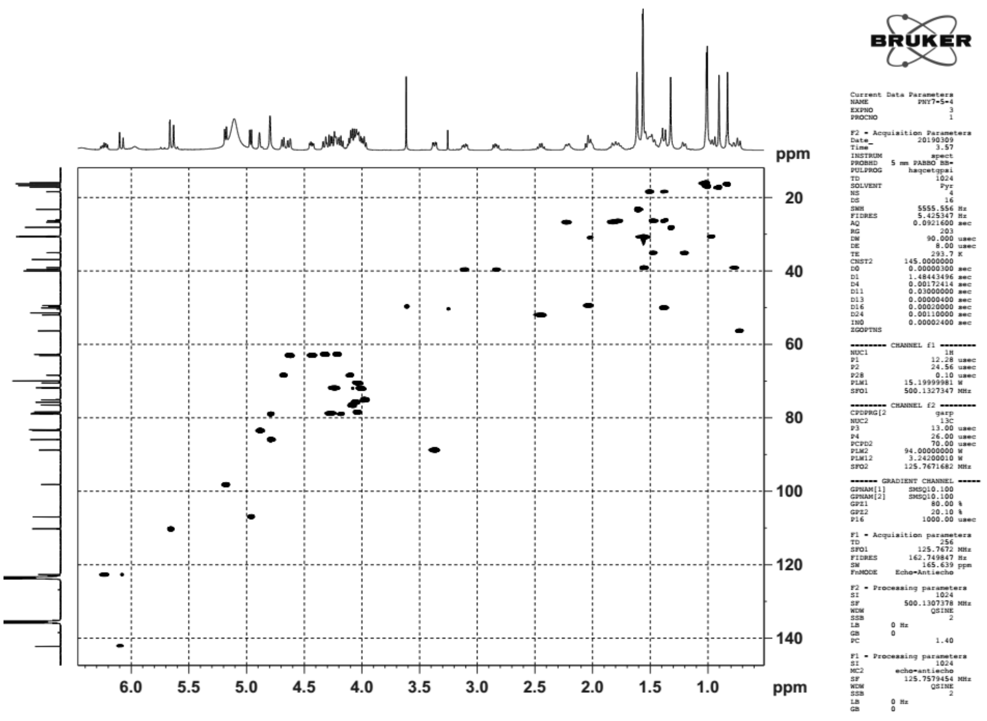

Figure S32 HSQC (C_5D_5N) spectrum of **5**

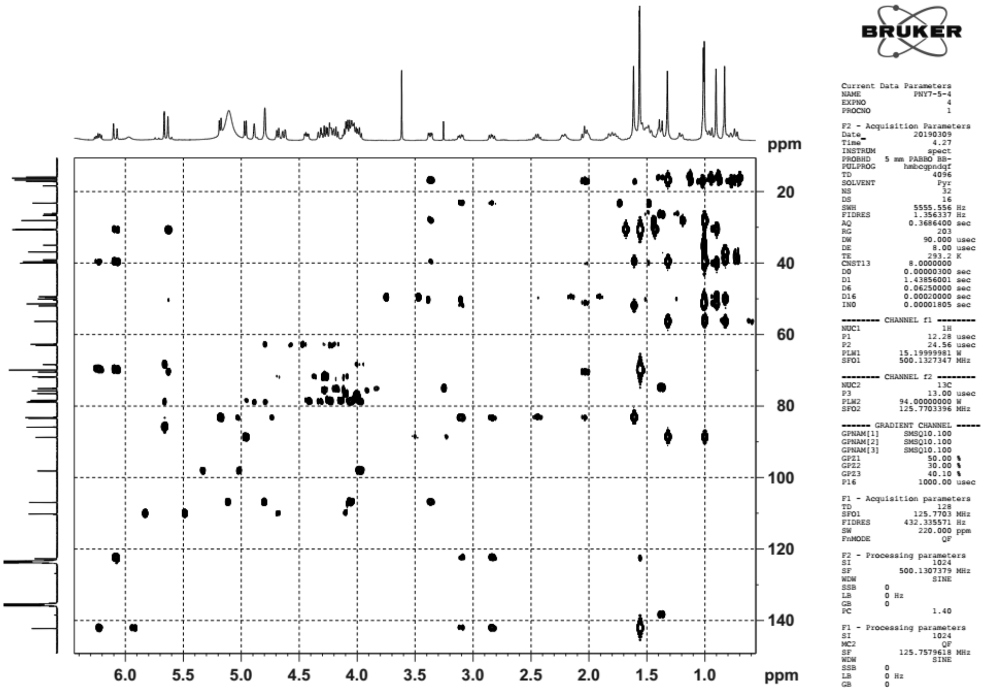

Figure S33 HMBC (C_5D_5N) spectrum of **5**

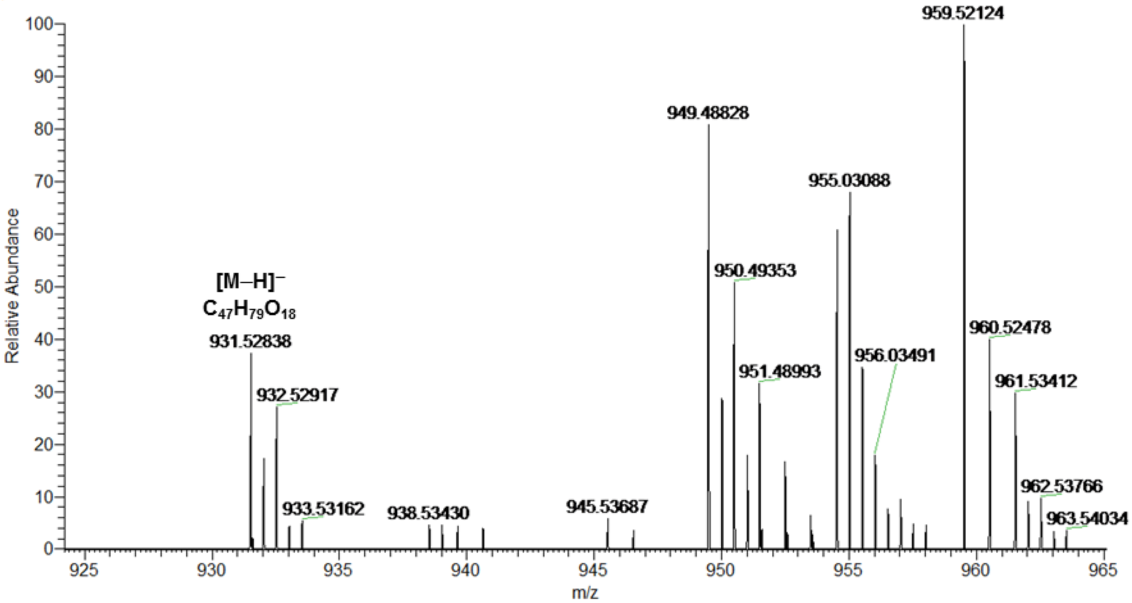

Figure S34 ESI-Q-Orbitrap-MS spectrum of **5**

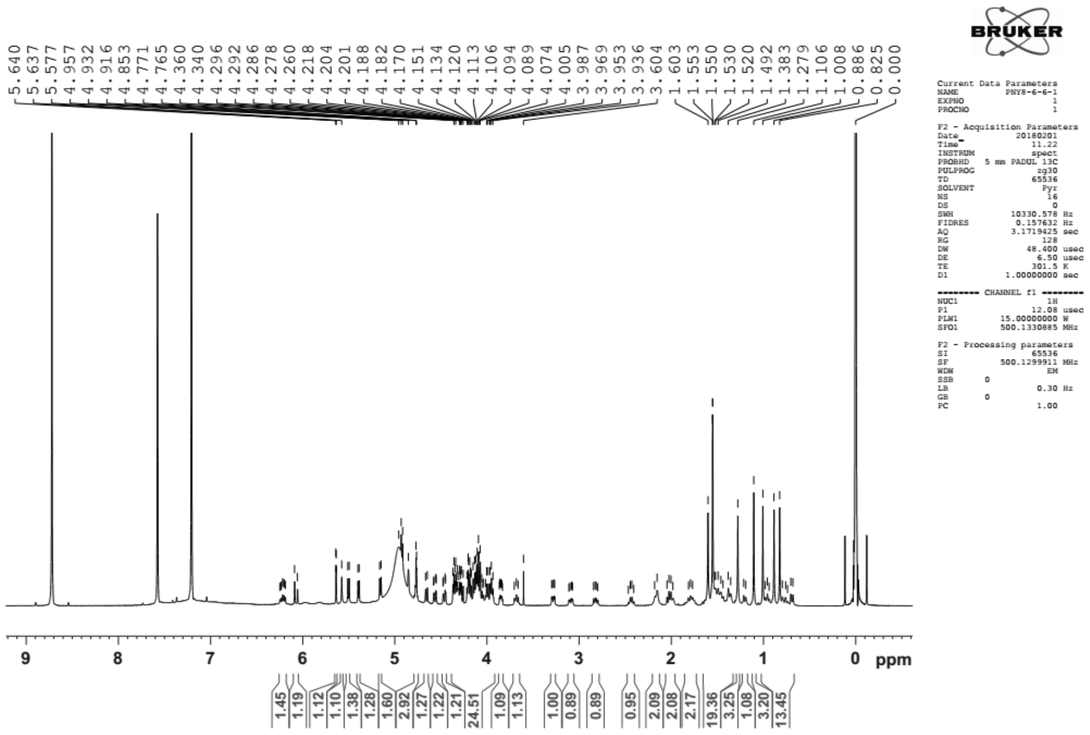

Figure S35 ^{1}H NMR (500 MHz, C_5D_5N) spectrum of **6**

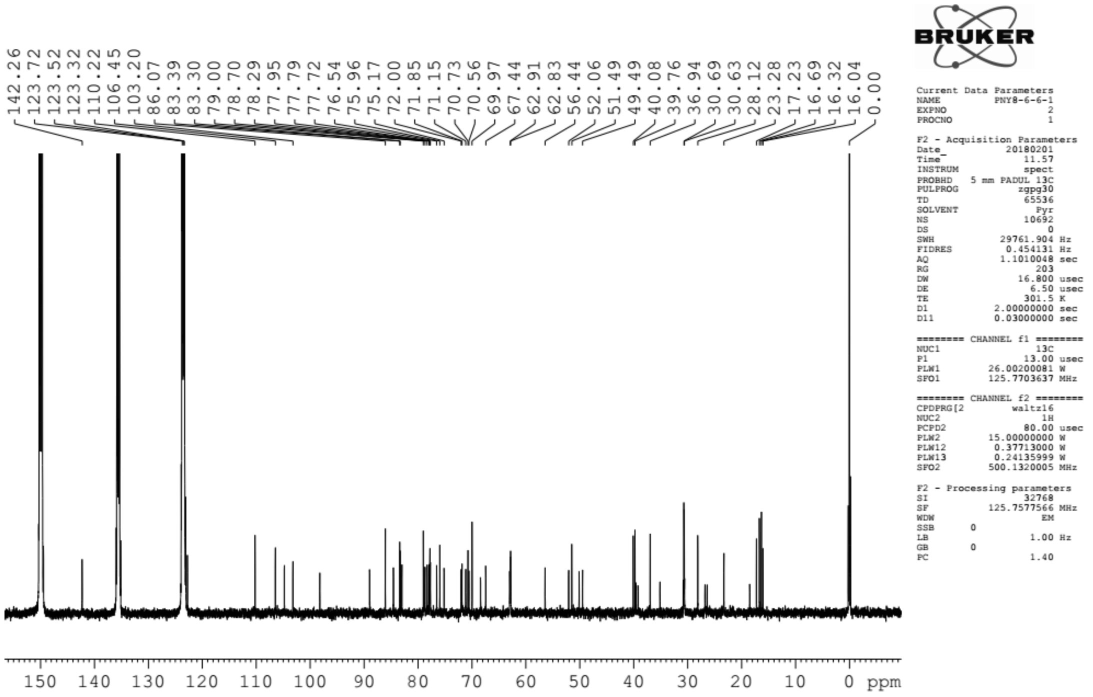

Figure S36 ^{13}C NMR (125 MHz, C_5D_5N) spectrum of **6**

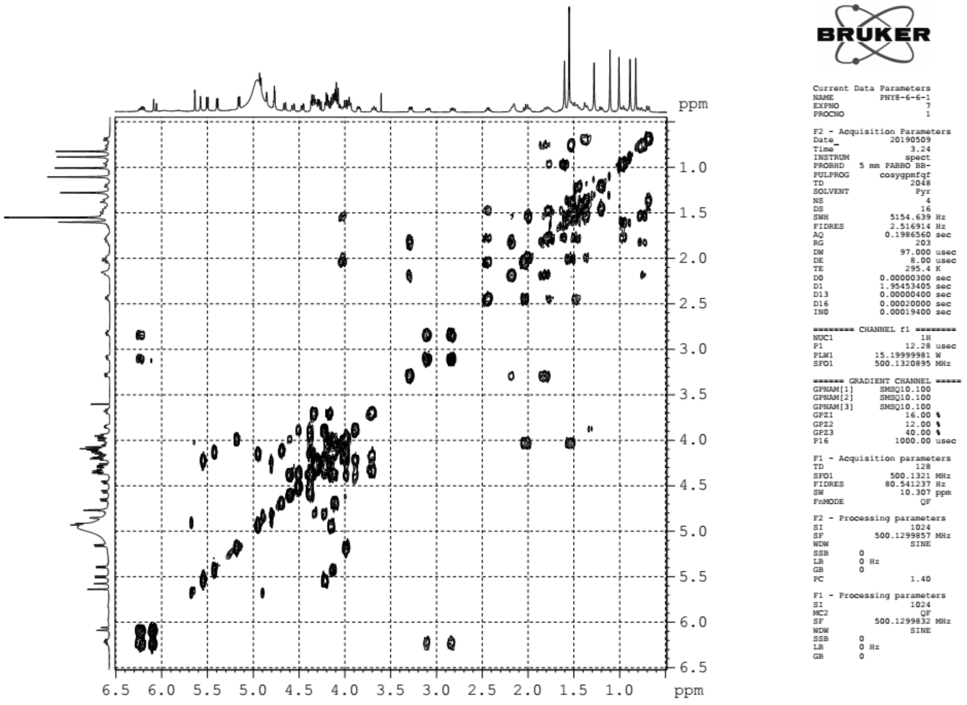

Figure S37 ^{1}H ^{1}H COSY (C_5D_5N) spectrum of **6**

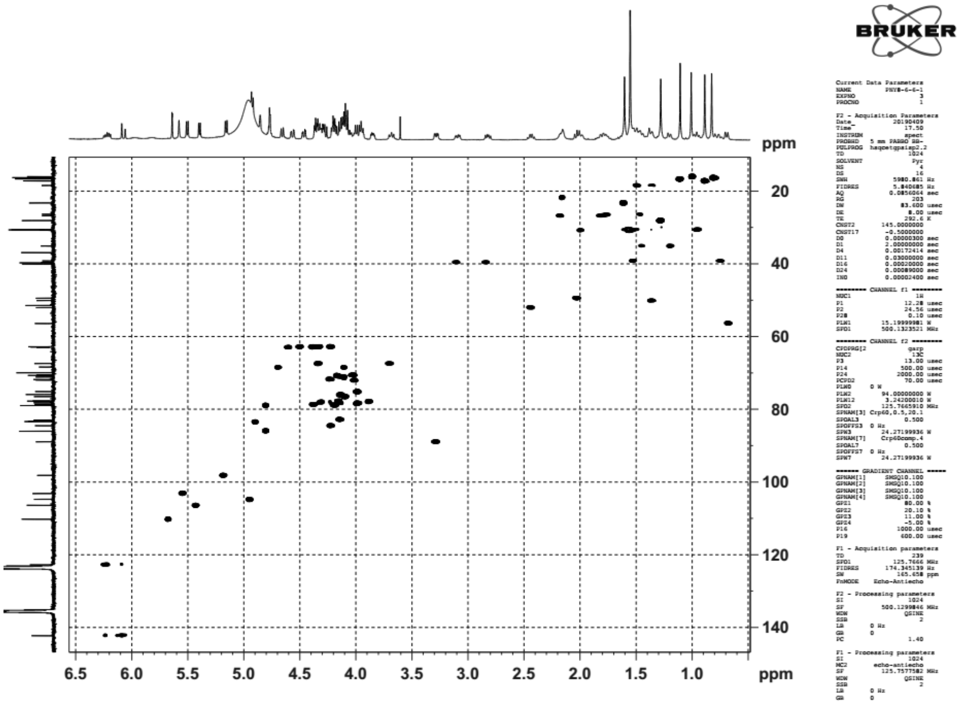

Figure S38 HSQC (C_5D_5N) spectrum of **6**

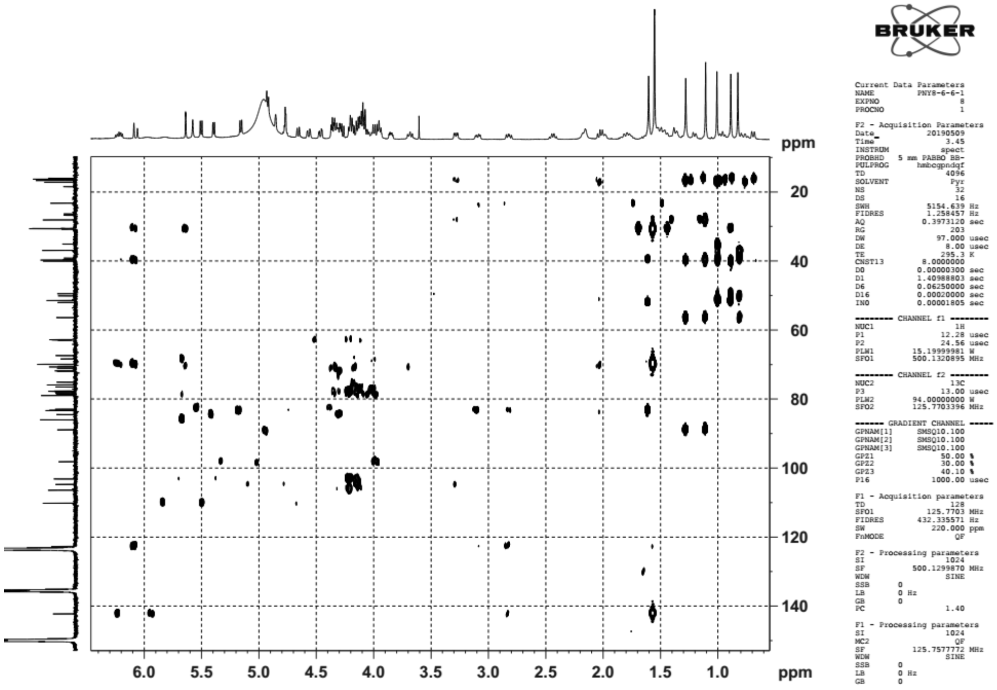

Figure S39 HMBC (C_5D_5N) spectrum of **6**

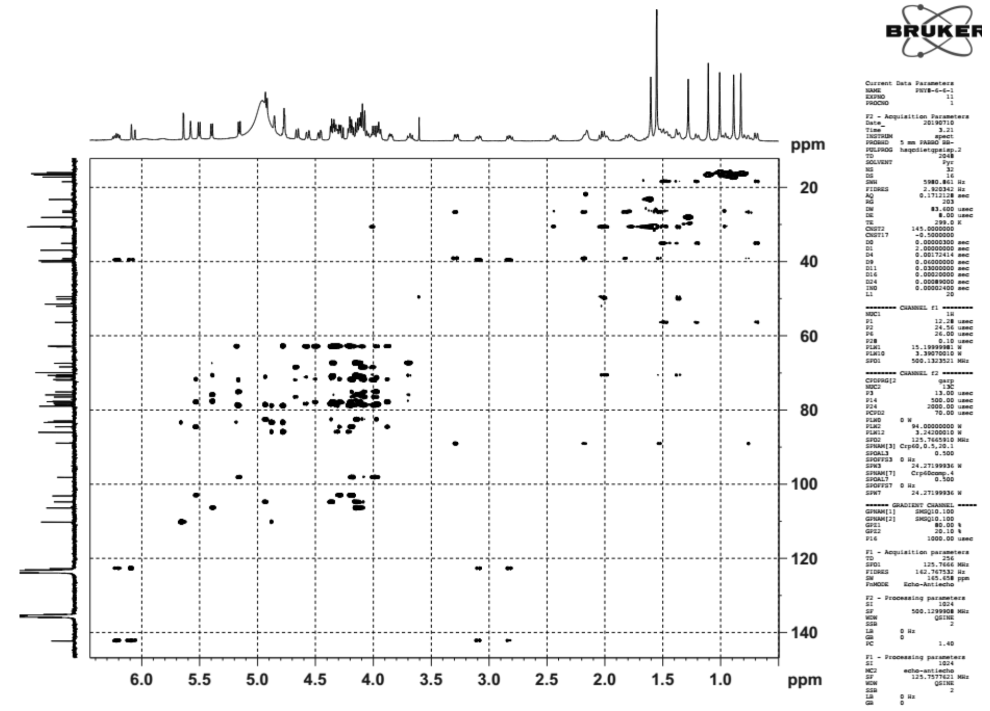

Figure S40 HSQC-TOCSY (C_5D_5N) spectrum of **6**

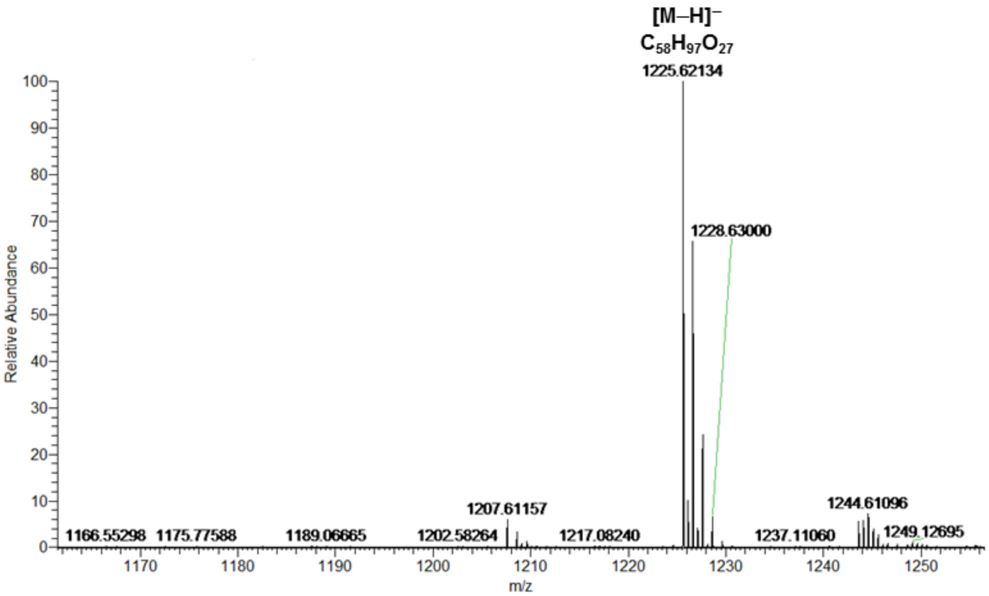

Figure S41 ESI-Q-Orbitrap-MS spectrum of **6**

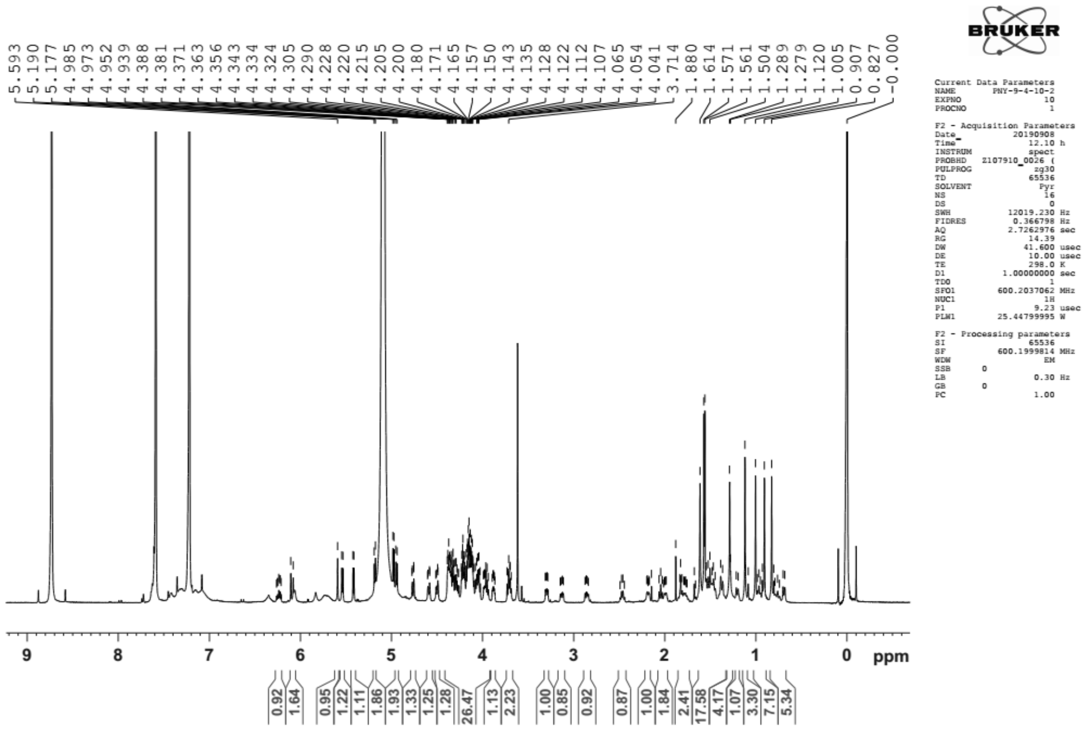

Figure S42 ^{1}H NMR (600 MHz, C_5D_5N) spectrum of **7**

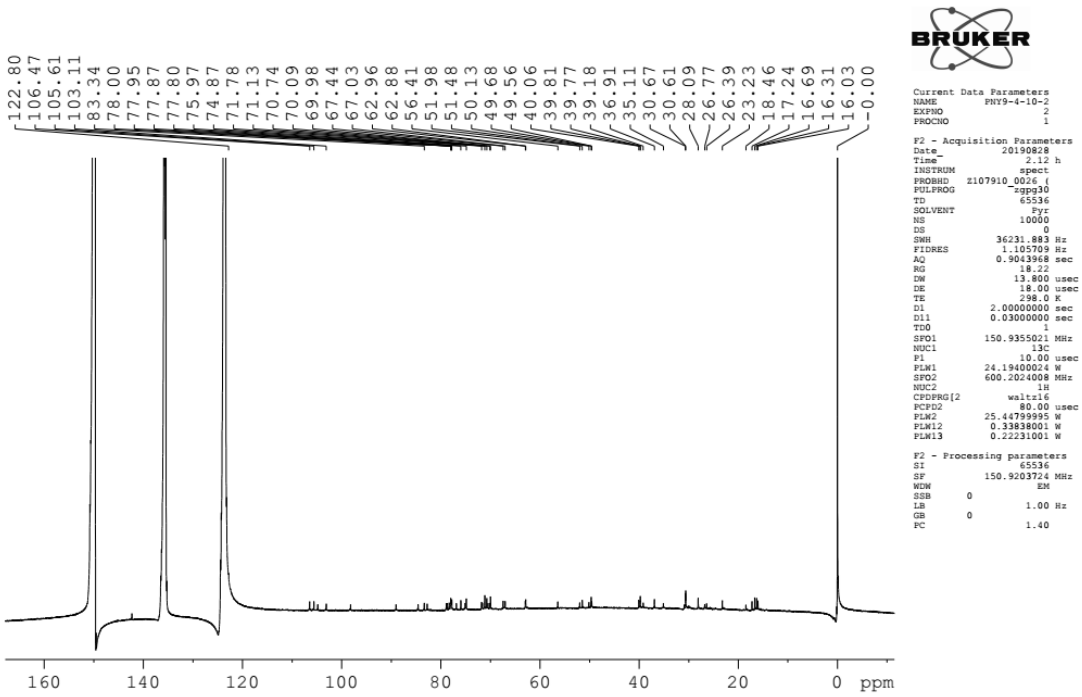

Figure S43 ^{13}C NMR (150 MHz, C_5D_5N) spectrum of **7**

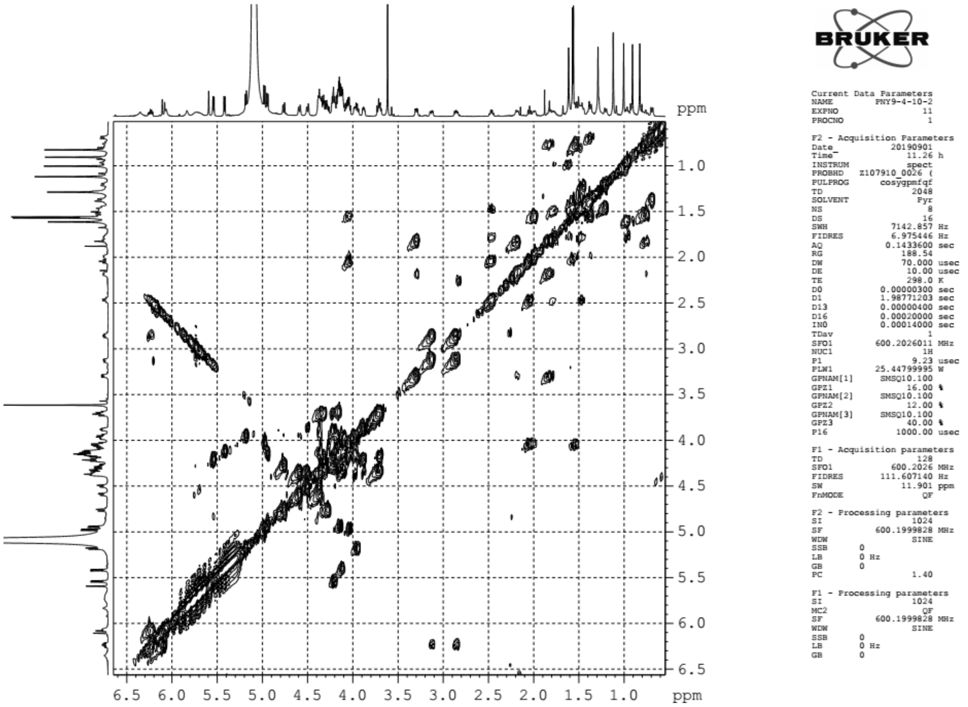

Figure S44 ^{1}H ^{1}H COSY (C_5D_5N) spectrum of **7**

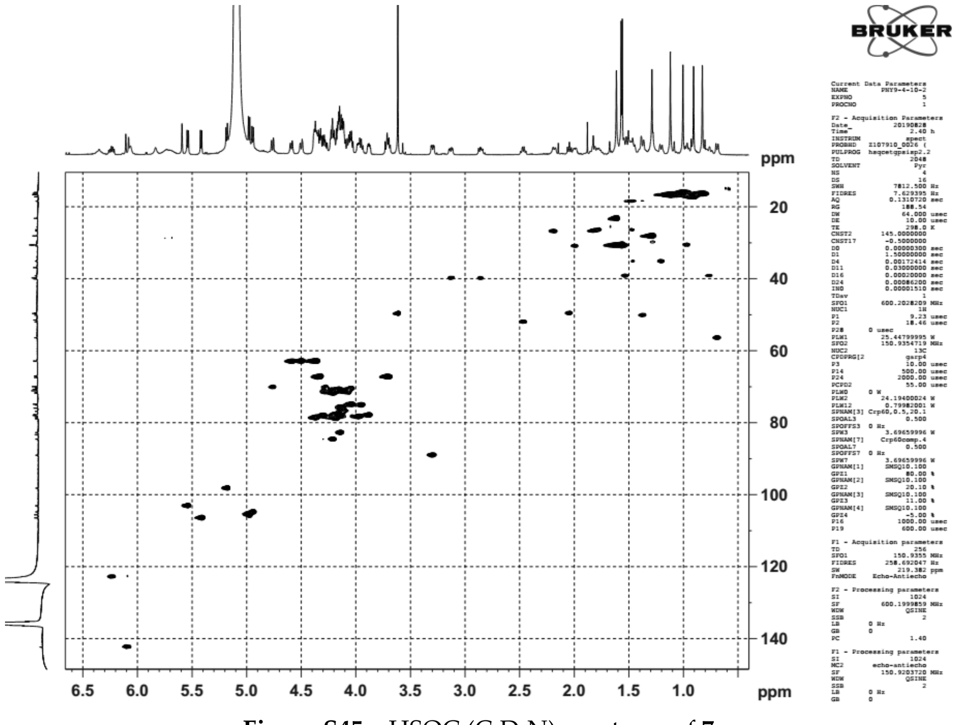

Figure S45 HSQC (C_5D_5N) spectrum of **7**

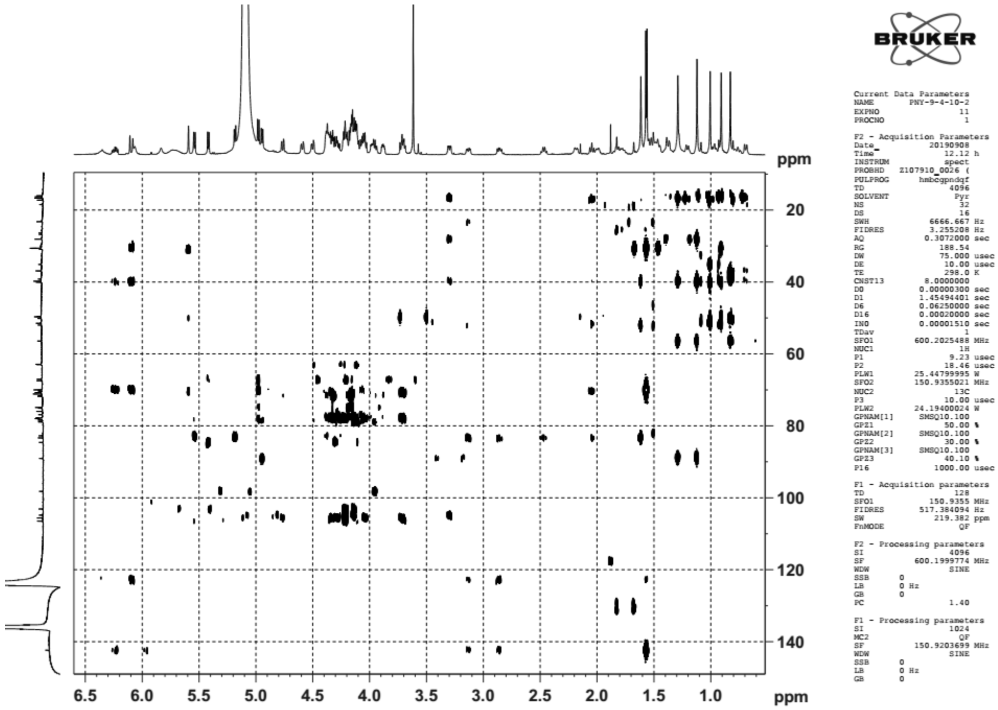

Figure S46 HMBC (C_5D_5N) spectrum of **7**

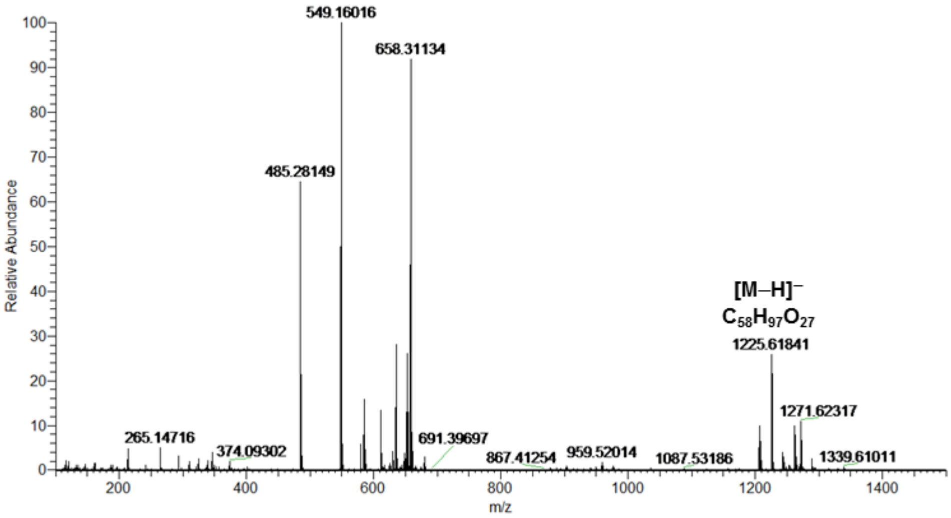

Figure S47 ESI-Q-Orbitrap-MS spectrum of **7**

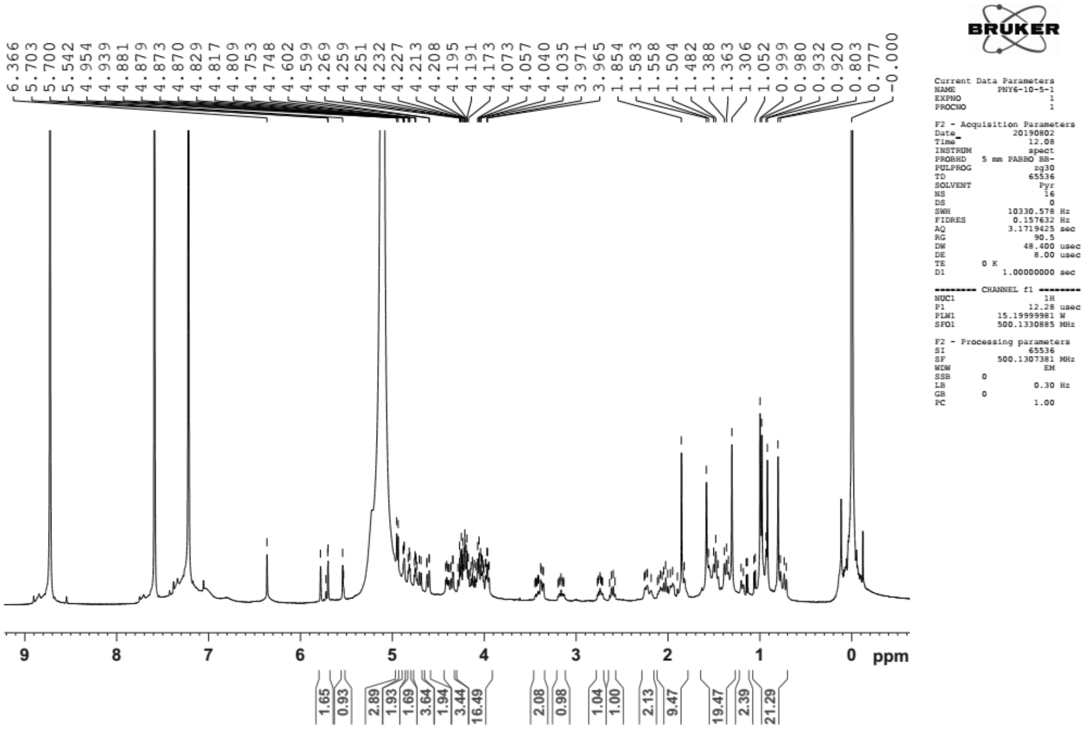

Figure S48 ^{1}H NMR (500 MHz, C_5D_5N) spectrum of **8**

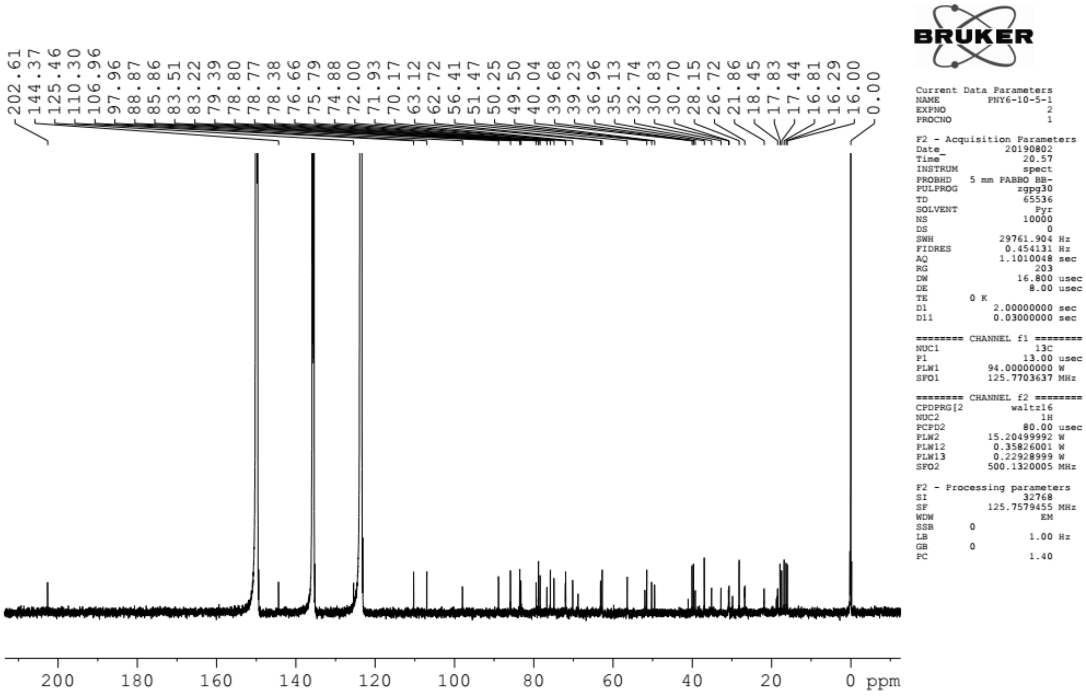

Figure S49 ^{13}C NMR (125 MHz, C_5D_5N) spectrum of **8**

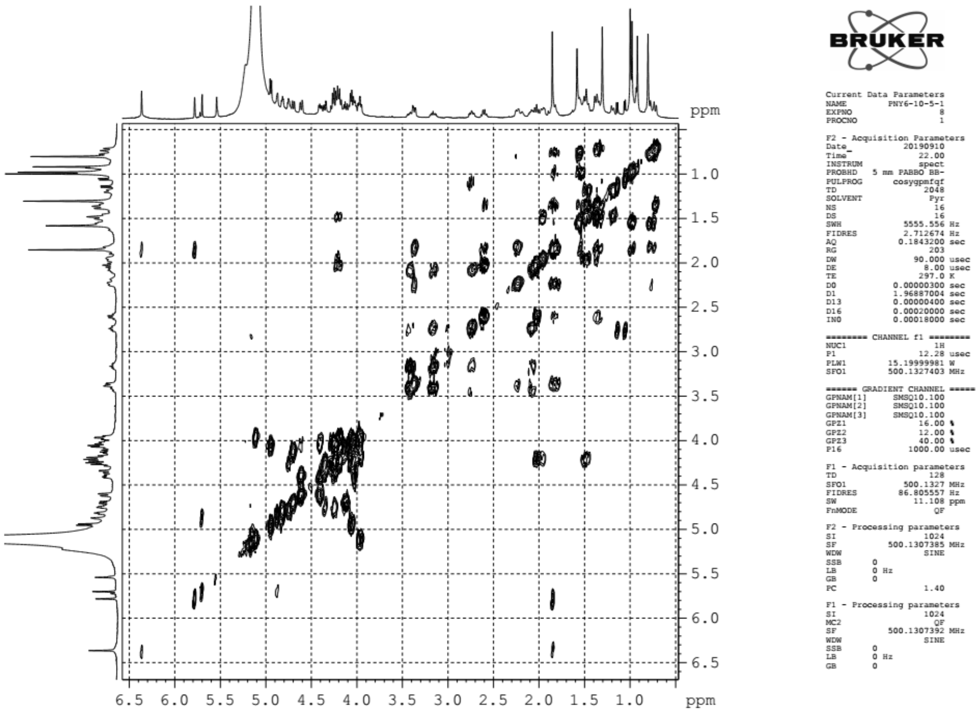

Figure S50 ^{1}H ^{1}H COSY (C_5D_5N) spectrum of **8**

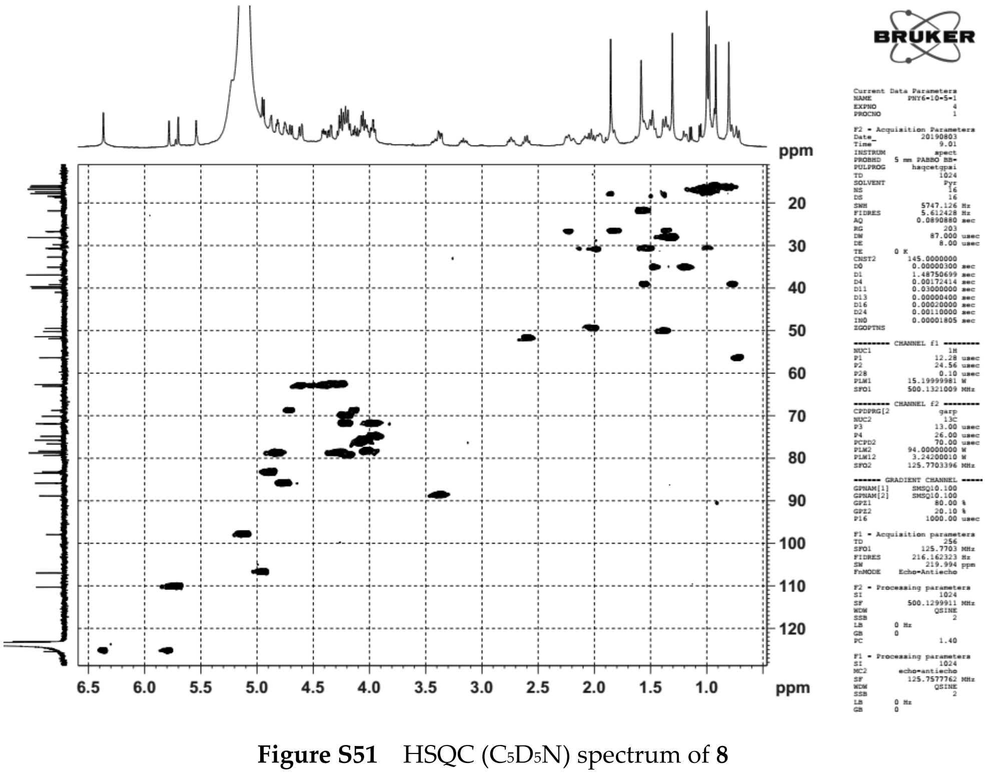

Figure S51 HSQC (C_5D_5N) spectrum of **8**

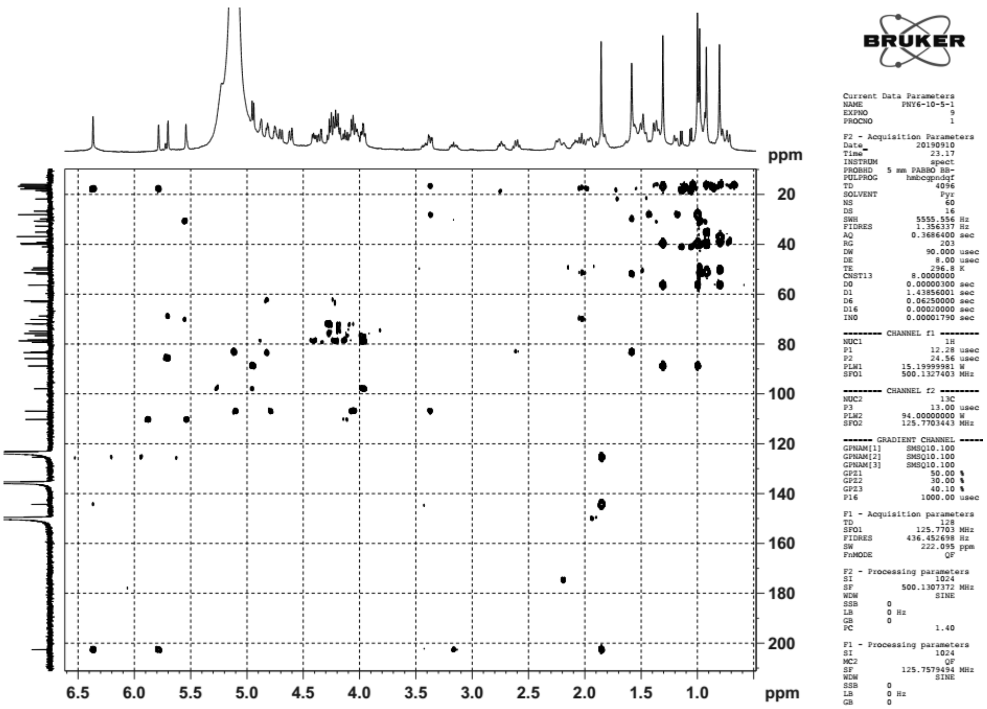

Figure S52 HMBC (C_5D_5N) spectrum of **8**

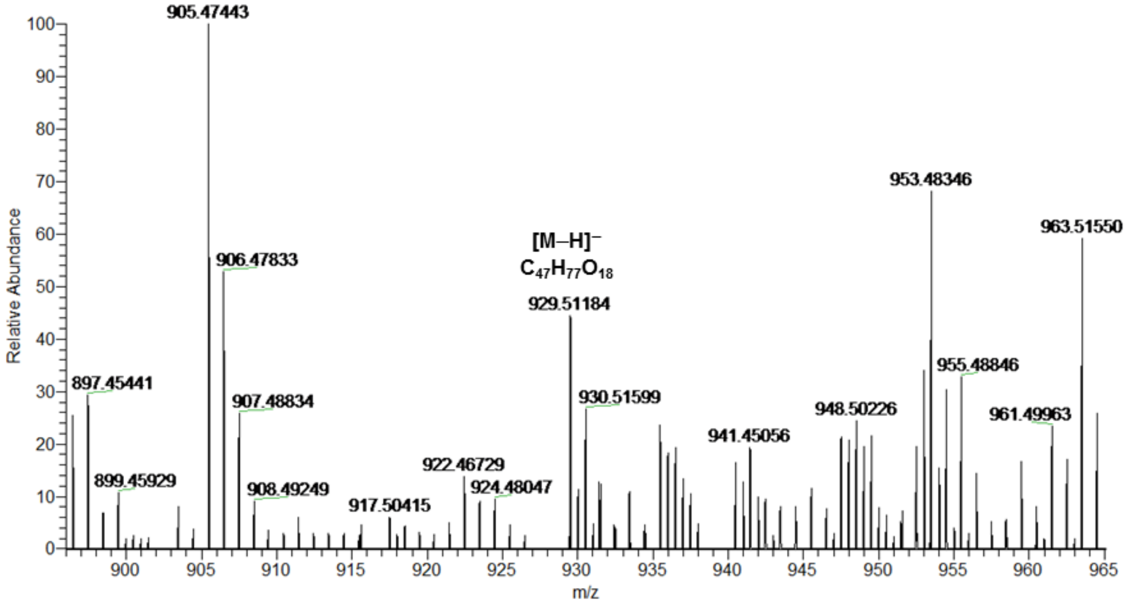

Figure S53 ESI-Q-Orbitrap-MS spectrum of **8**

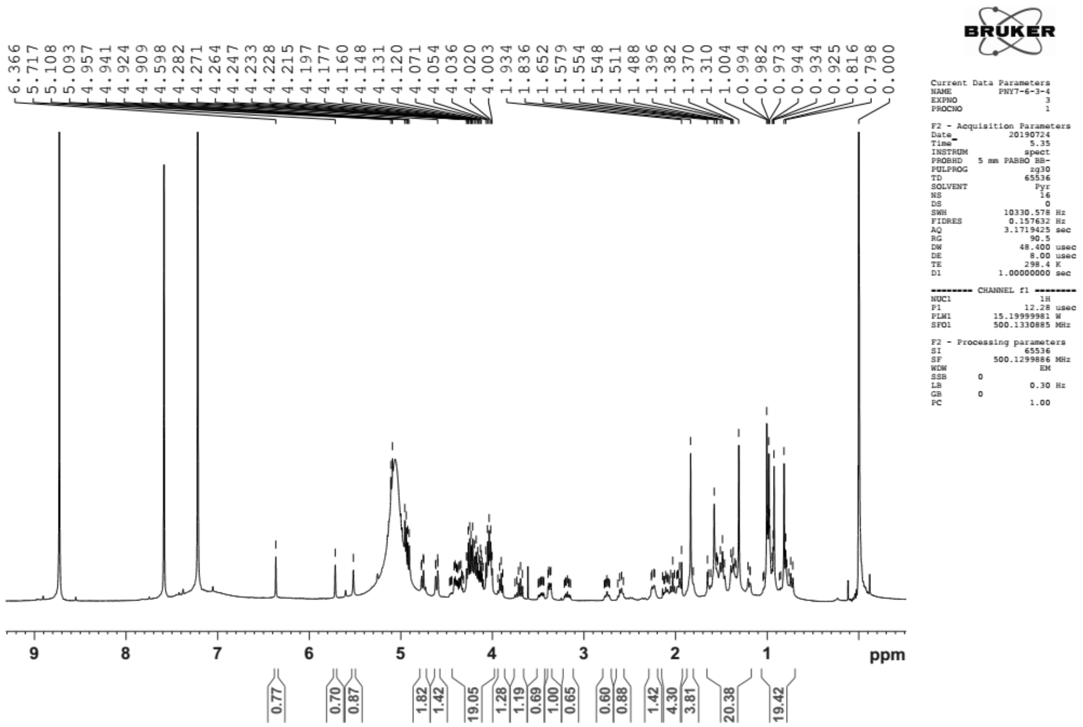

Figure S54 ^{1}H NMR (500 MHz, C_5D_5N) spectrum of **9**

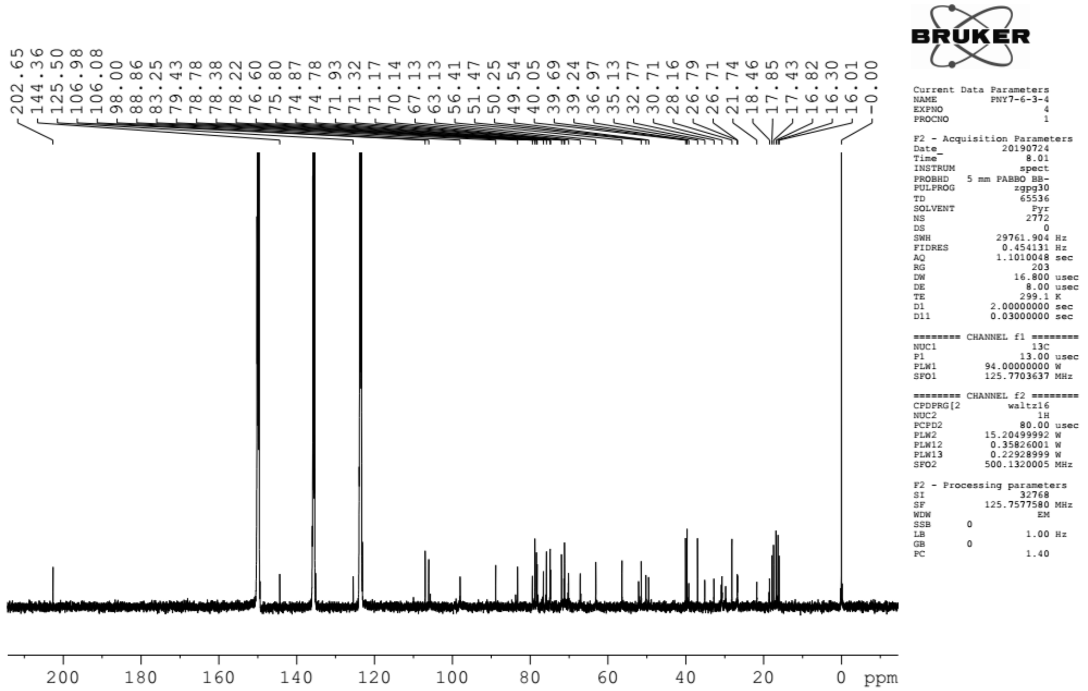

Figure S55 ^{13}C NMR (125 MHz, C_5D_5N) spectrum of **9**

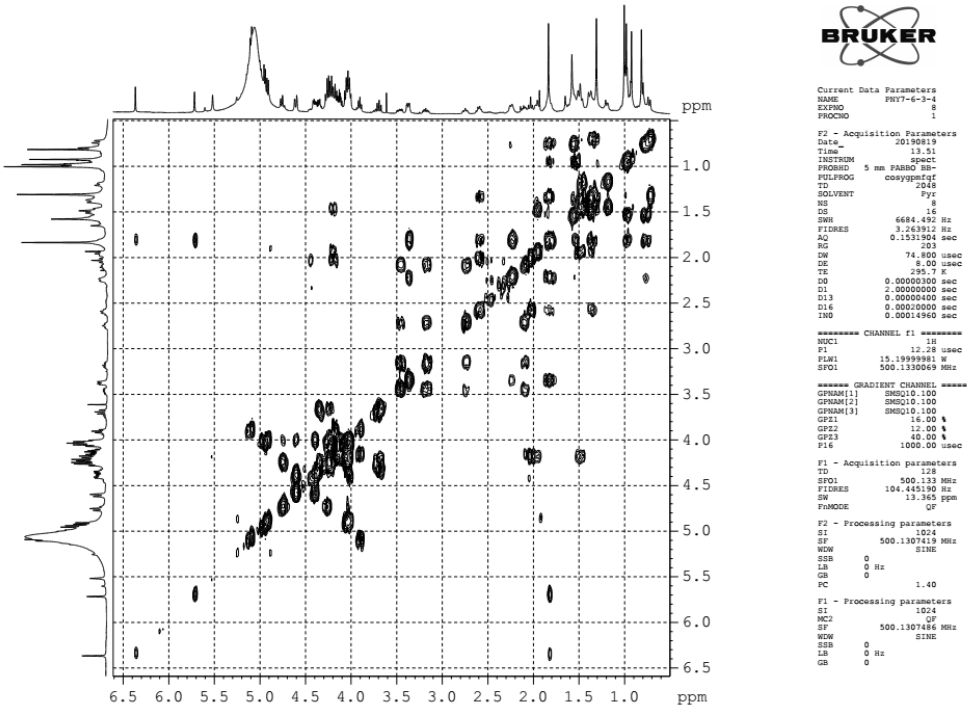

Figure S56 ^{1}H ^{1}H COSY (C_5D_5N) spectrum of **9**

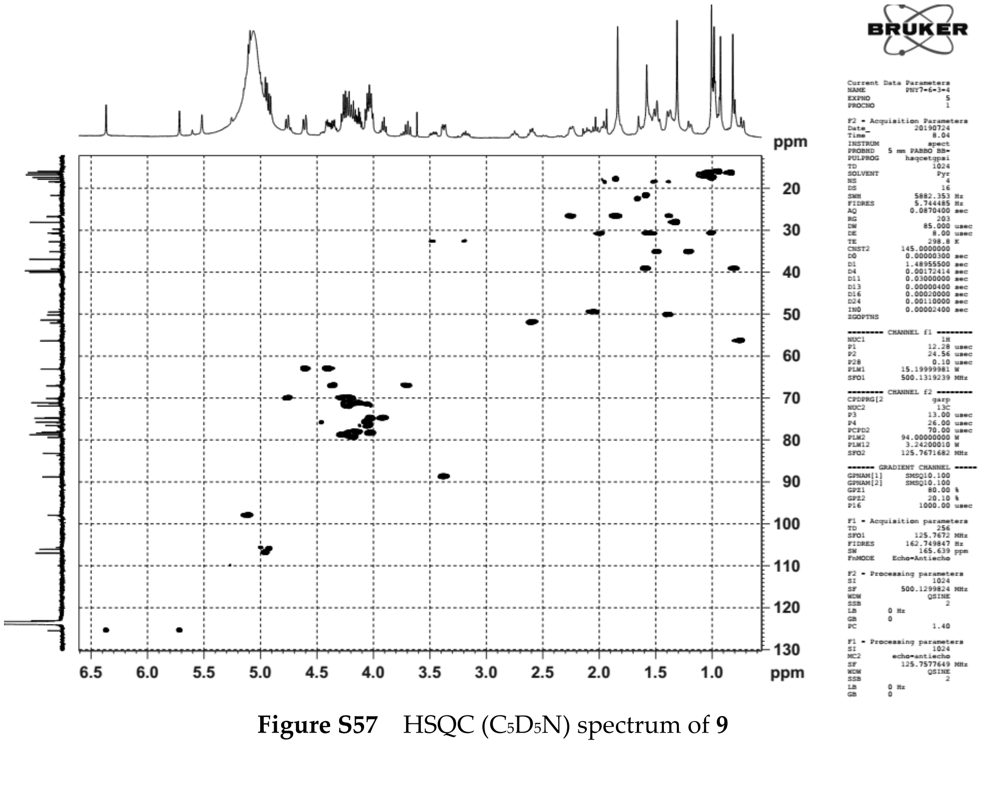

Figure S57 HSQC (C_5D_5N) spectrum of **9**

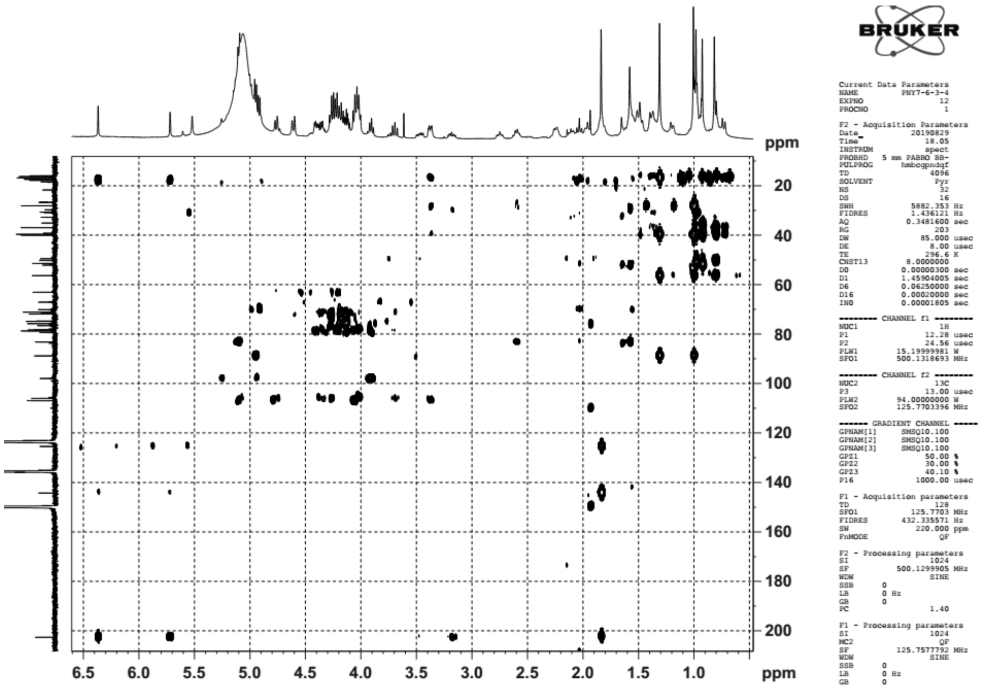

Figure S58 HMBC (C_5D_5N) spectrum of **9**

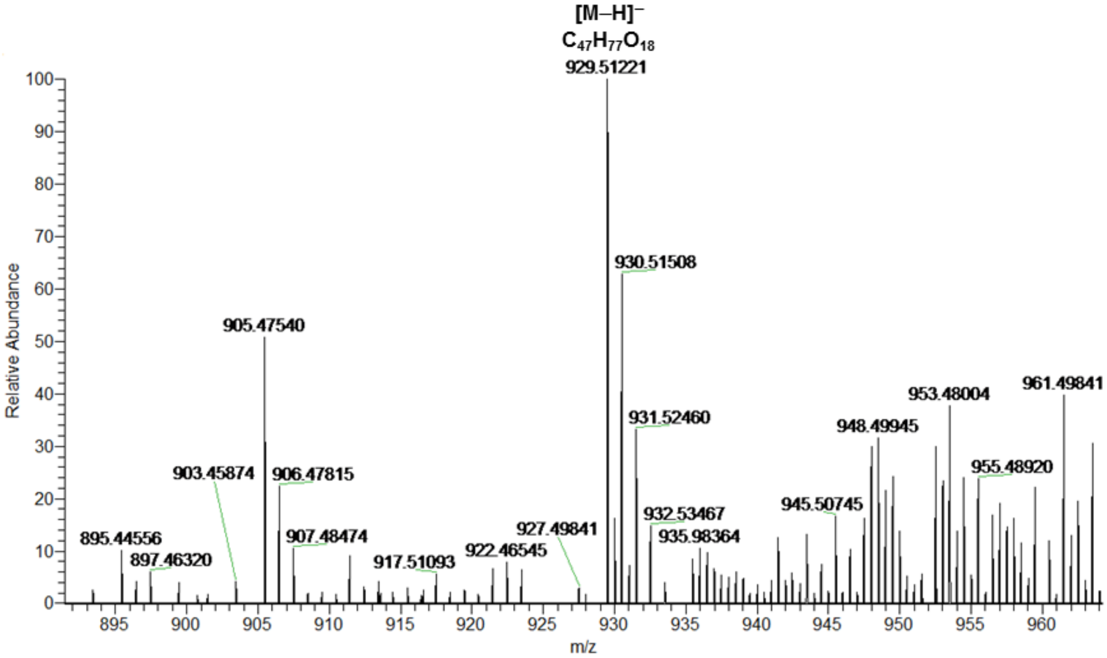

Figure S59 ESI-Q-Orbitrap-MS spectrum of **9**

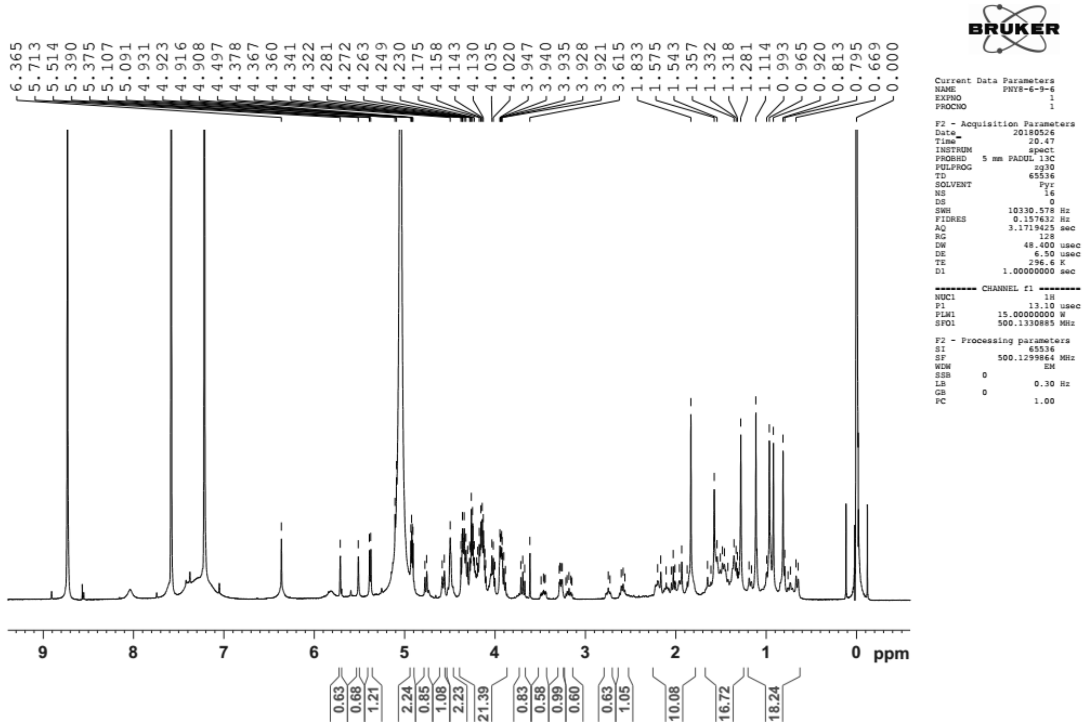

Figure S60 ^{1}H NMR (500 MHz, C_5D_5N) spectrum of PNY8-6-9-6 (**10**)

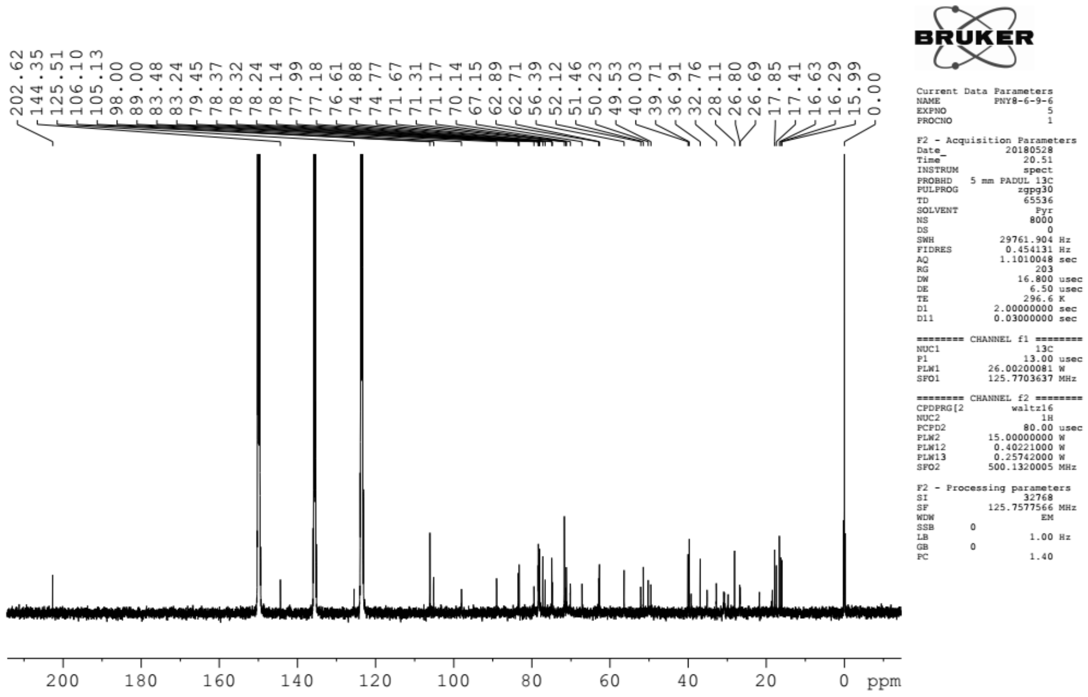

Figure S61 ^{13}C NMR (125 MHz, C_5D_5N) spectrum of **10**

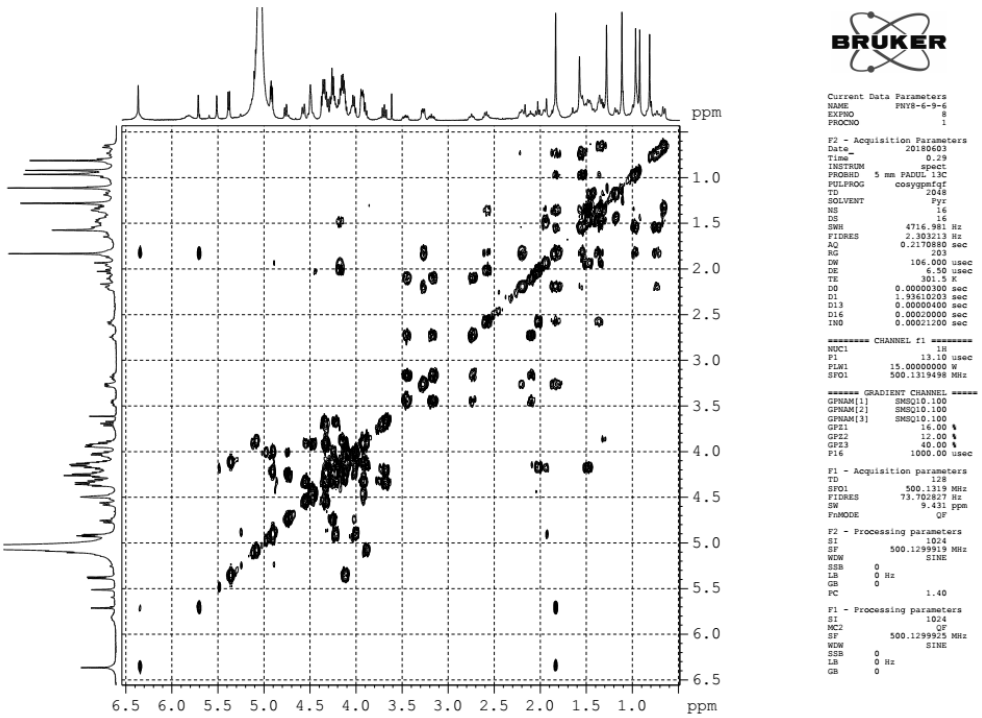

Figure S62 ^1H ^1H COSY (C_5D_5N) spectrum of **10**

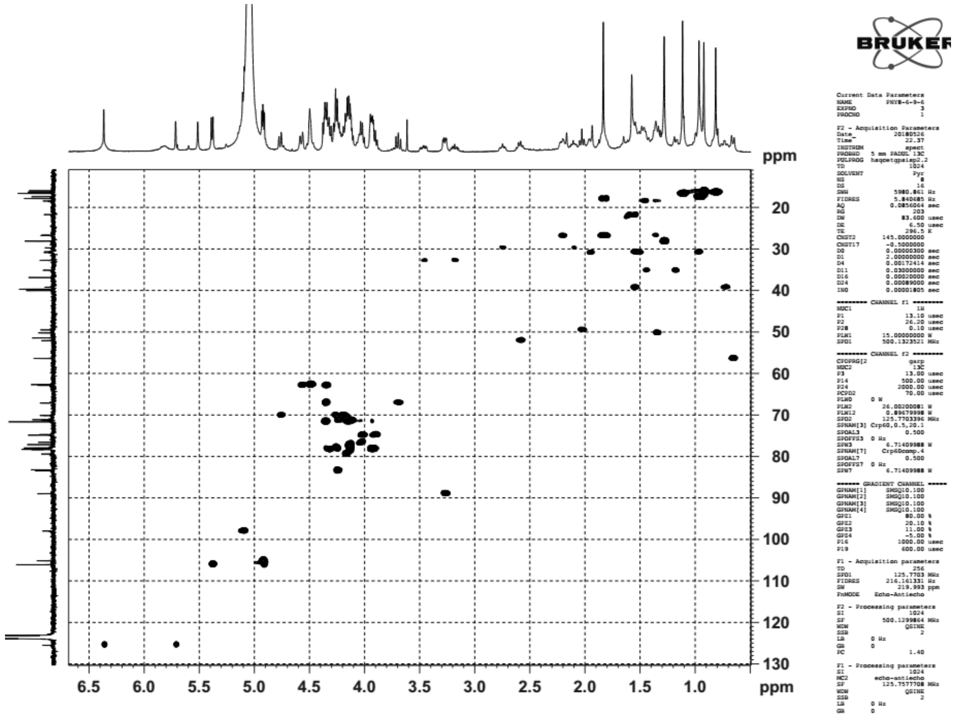

Figure S63 HSQC (C_5D_5N) spectrum of **10**

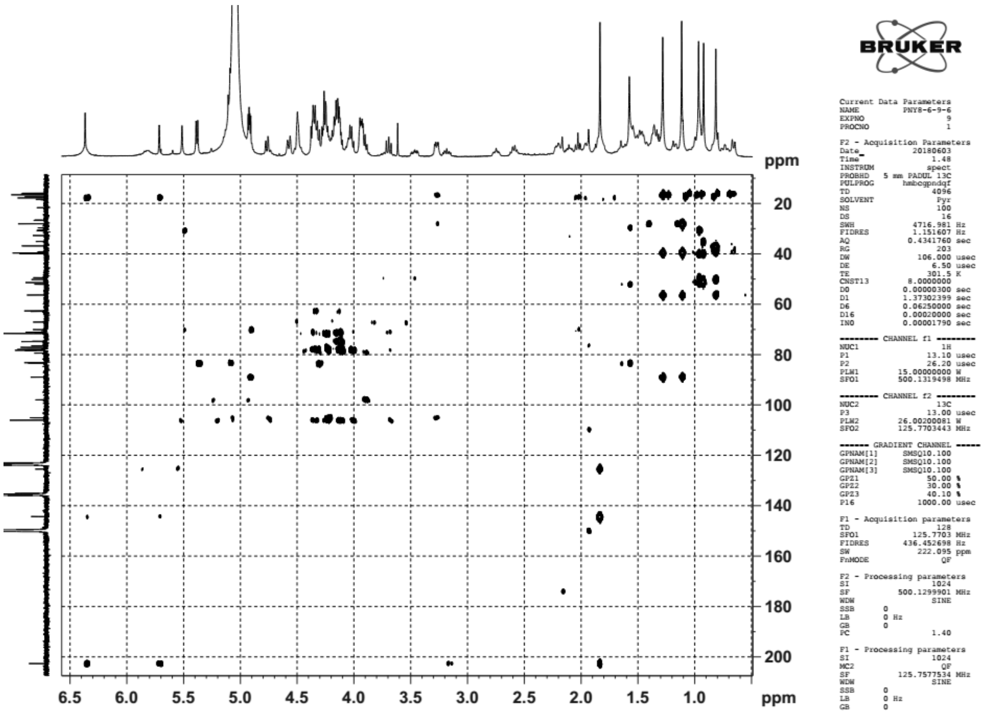

Figure S64 HMBC (C_5D_5N) spectrum of **10**

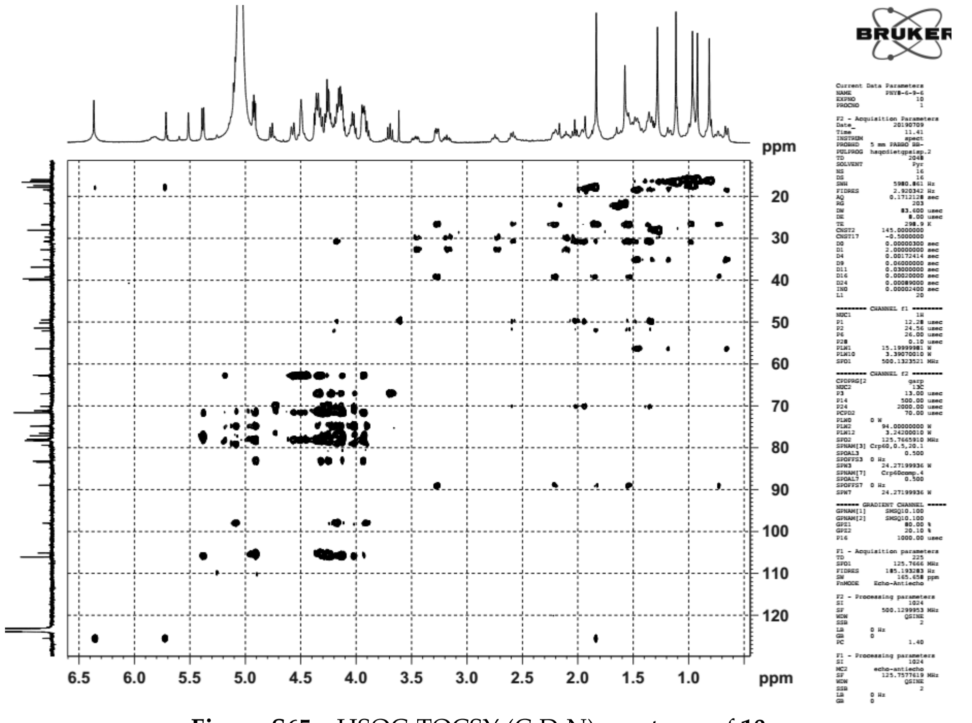

Figure S65 HSQC-TOCSY (C_5D_5N) spectrum of **10**

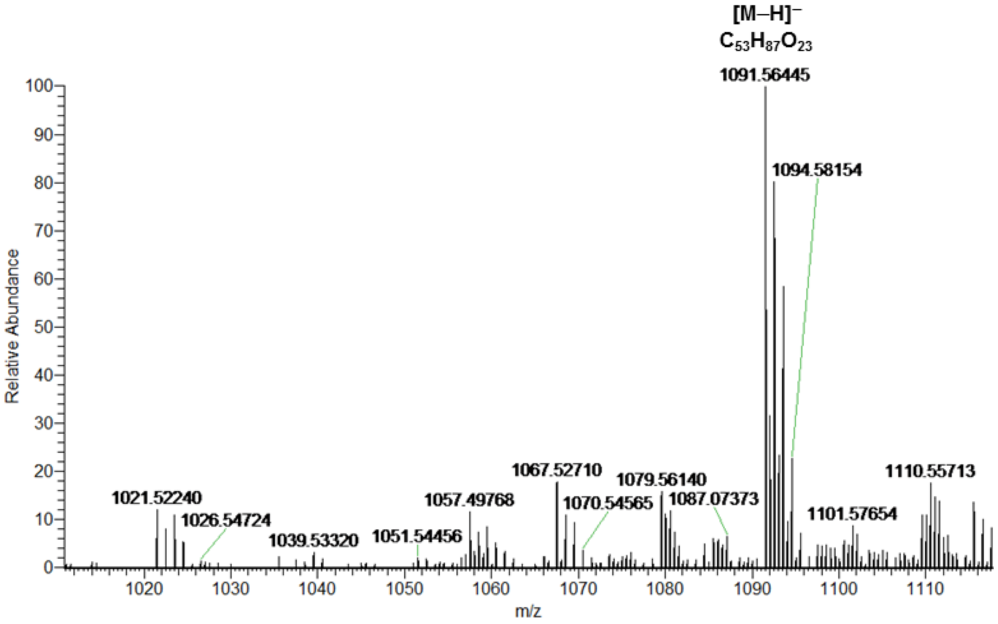

Figure S66 ESI-Q-Orbitrap-MS spectrum of **10**

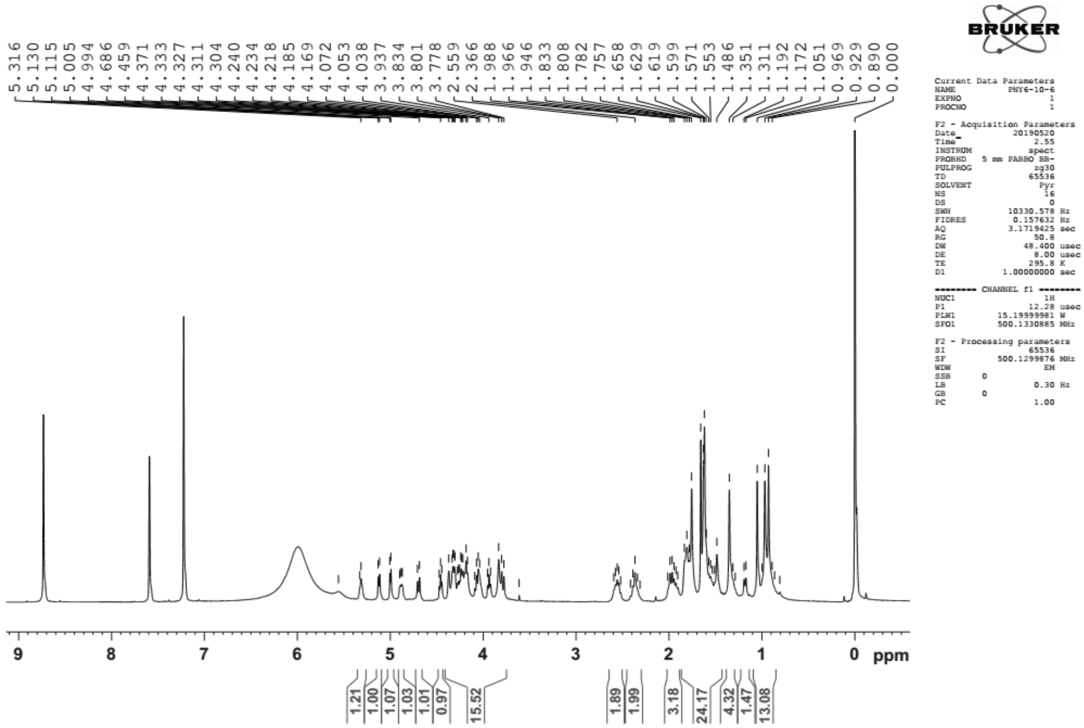

Figure S67 ^{1}H NMR (500 MHz, C_5D_5N) spectrum of **11**

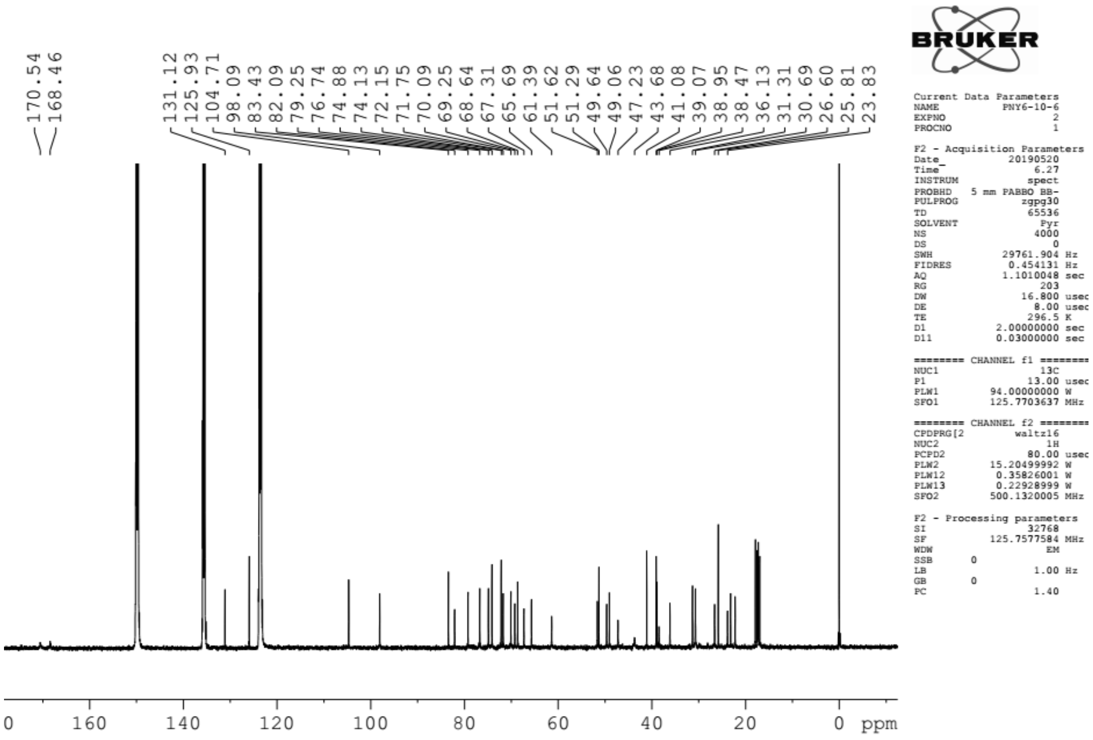

Figure S68 ^{13}C NMR (125 MHz, C_5D_5N) spectrum of **11**

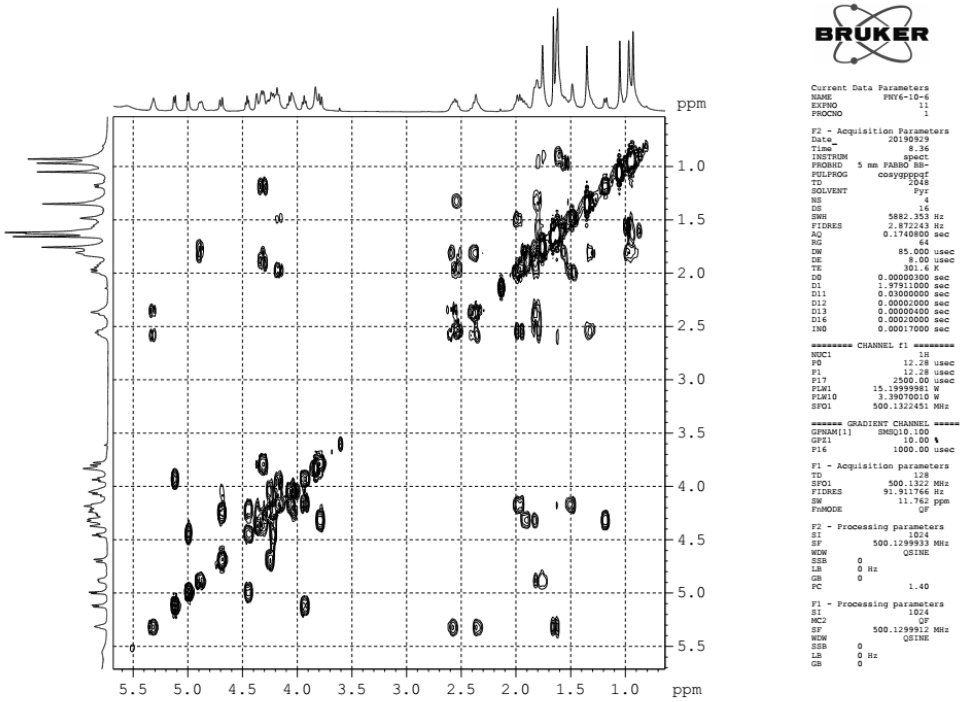

Figure S69 ^1H ^1H COSY (C_5D_5N) spectrum of **11**

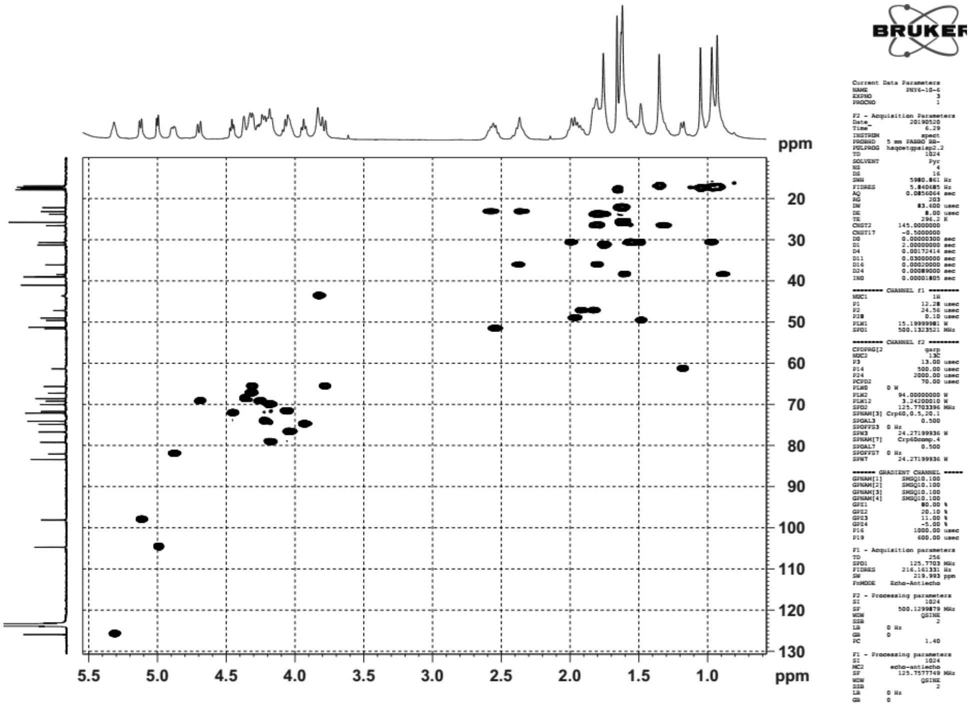

Figure S70 HSQC (C_5D_5N) spectrum of **11**

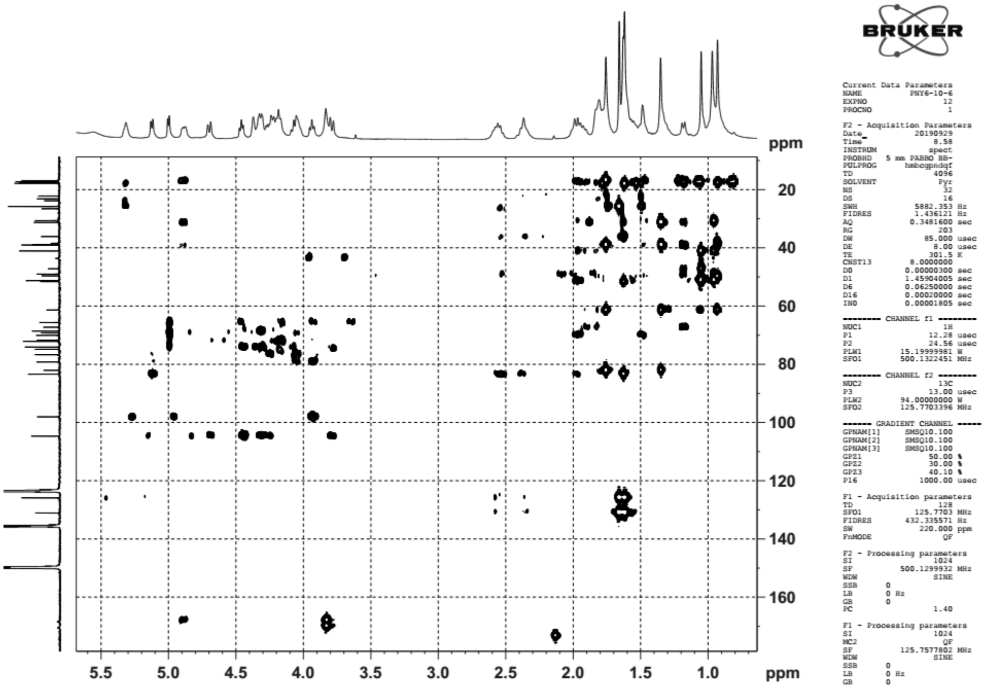

Figure S71 HMBC (C_5D_5N) spectrum of **11**

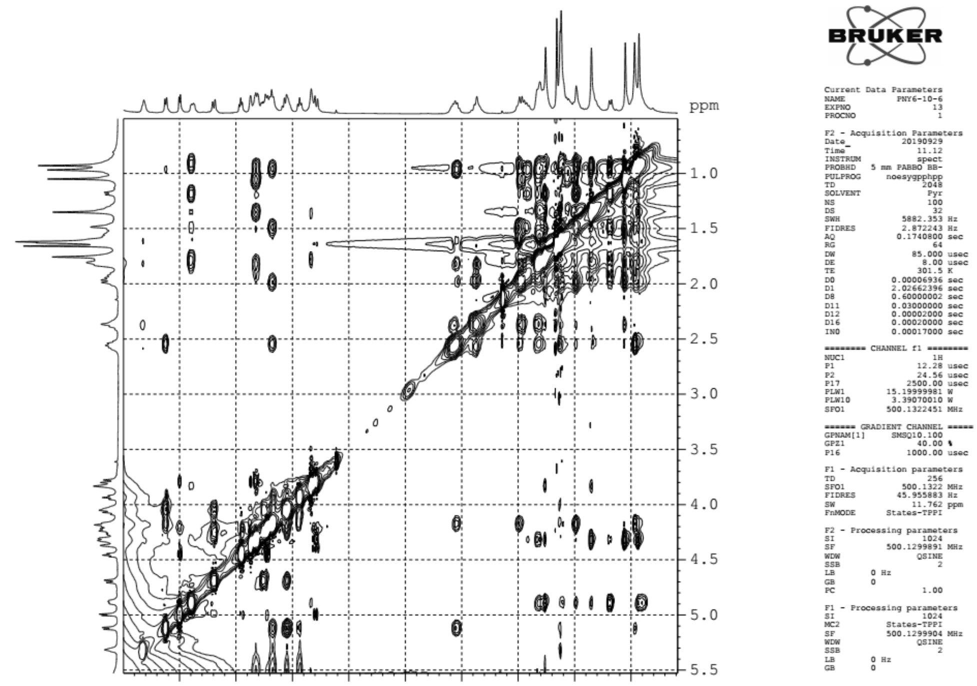

Figure S72 NOSEY (C_5D_5N) spectrum of **11**

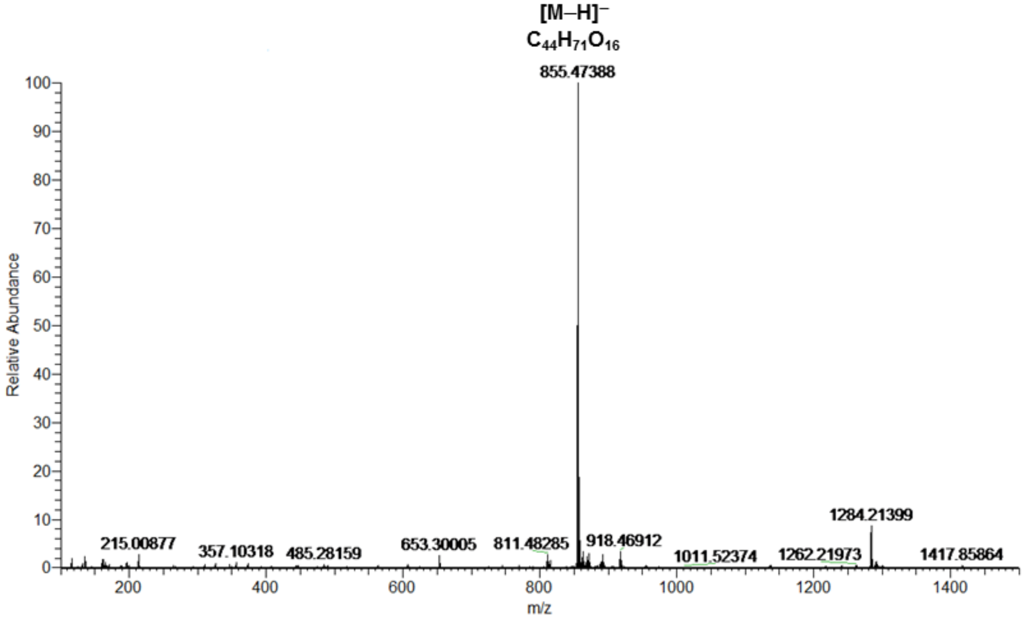

Figure S73 ESI-Q-Orbitrap-MS spectrum of **11**

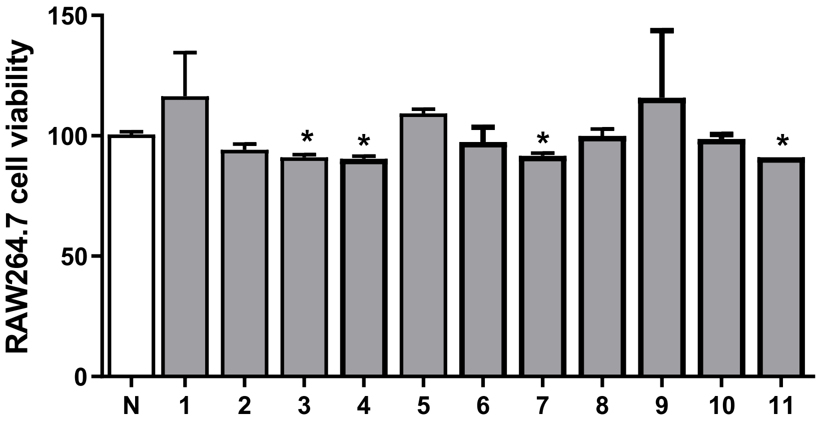

Figure S74 MTT assay of compounds **1–11** at the concentration of 50 μM
